# Towards Assessing the Human Trajectory Planning Horizon

**DOI:** 10.1371/journal.pone.0167021

**Published:** 2016-12-09

**Authors:** Daniel Carton, Verena Nitsch, Dominik Meinzer, Dirk Wollherr

**Affiliations:** 1 Chair of Automatic Control Engineering, Technical University of Munich, Theresienstr. 90, 80333 Munich, Germany; 2 Human Factors Institute, Universität der Bundeswehr München, Werner-Heisenberg-Weg 39, 85577 Neubiberg, Germany; Politecnico University of Bucharest, ROMANIA

## Abstract

Mobile robots are envisioned to cooperate closely with humans and to integrate seamlessly into a shared environment. For locomotion, these environments resemble traversable areas which are shared between multiple agents like humans and robots. The seamless integration of mobile robots into these environments requires accurate predictions of human locomotion. This work considers optimal control and model predictive control approaches for accurate trajectory prediction and proposes to integrate aspects of human behavior to improve their performance. Recently developed models are not able to reproduce accurately trajectories that result from sudden avoidance maneuvers. Particularly, the human locomotion behavior when handling disturbances from other agents poses a problem. The goal of this work is to investigate whether humans alter their trajectory planning horizon, in order to resolve abruptly emerging collision situations. By modeling humans as model predictive controllers, the influence of the planning horizon is investigated in simulations. Based on these results, an experiment is designed to identify, whether humans initiate a change in their locomotion planning behavior while moving in a complex environment. The results support the hypothesis, that humans employ a shorter planning horizon to avoid collisions that are triggered by unexpected disturbances. Observations presented in this work are expected to further improve the generalizability and accuracy of prediction methods based on dynamic models.

## Introduction

A wide variety of robotic applications has emerged recently. Sensor driven driver assistance systems are commonly available and vacuum cleaner robots already have a strong position in the consumer electronics market. Autonomous cars and trucks are being developed to take over in traffic on highways, where they are required to progress in the planned journey while safely moving amongst other human steered cars. Robots that carry supplies to workstations and replenish parts for assembly at a production line are being deployed at factories alongside automated forklifts. With the additional upgrowth of industry 4.0, many applications arise where robots could share a workspace with humans. Current trends aspire mobile robots that deliver packages in cities and move freely in factories or warehouses along with humans. Robots deployed in the logistics sector for fulfillment tasks directly share a workspace with humans during locomotion and picking. Furthermore, collaborative assembly tasks are envisioned, where one or multiple intelligent robot arms assist a human by providing tools or parts.

These tasks require robots to integrate seamlessly into these environments. When multiple agents, meaning robots and humans, must traverse a shared environment in a seamless manner, mutual prediction is a key ability. Therefore, robots must be able to accurately predict human locomotion. Accurate predictions allow a robotic system to move seamlessly among the surrounding interaction partners and to minimize disturbances since collisions are avoided prematurely. This ability benefits the applications since a are more efficient and convenient collaboration and interaction is enabled [1, 2]. Thus, understanding human motion planning will allow us to improve prediction methods and raise the efficiency of robots in shared workspaces.

Numerous approaches for motion prediction are already available [3–6]. State-of-the-Art methods are based on learning approaches or use dynamic models which approximate the human musculosceletal system. Machine learning methods usually consider fully observable environments with features that determine the motion and thus allow for predictions. These approaches yield a probability for a human to occupy a certain position [6]. Methods based on dynamic models mostly apply optimal control methods, where specifically designed objective functions lead to trajectories that closely resemble human locomotion [4, 7–9]. An advantage of the latter approaches is the resulting continuous trajectory, which describes all attributes of a motion from positions down to torques.

Yet, efficient and reliable prediction over a large horizon is still an ongoing research topic. Especially the varying collision avoidance behaviors of humans pose a challenging aspect for optimal control based methods. Current models for human locomotion do not generalize to the wide variety of observed situations and the respective human behaviors. Literature shows, that current models are especially not able to accurately represent the observed trajectories from a moving human that is disturbed by another agent [4, 10]. These disturbances are unexpected events, e.g. due to uncertainty or prediction errors, that influence the agent’s path. They lead to specific avoidance or recovery behaviors, i.e. short-term reactions with sudden path or velocity adaptations, and result in suboptimal trajectories. As this is not covered by the models, they are not able to produce a suitable trajectory prediction. Especially, research towards collision avoidance behaviors encounters this problem [4, 10, 11]. It is reported that the applied optimal control approaches do not resemble the observed behaviors well [4, 10]. Obviously, the objective functionals of the methods, which are usually driven by energy minimization, curvature constraints or velocity adjustments, do not cover these short-term behaviors.

Our work addresses this problem and aims to identify human locomotion behaviors that account for these situations and have the potential to enhance prediction algorithms for robotic applications in shared environments. Previous research demonstrated, that the consideration of human behaviors can significantly improve the performance of tracking and prediction methods [12]. Specifically optimal control and model predictive control (MPC) methods are to be improved [4, 10, 11]. The incorporation of the identified behaviors is anticipated to enable a prediction of human locomotion in cases where the initial optimal solution is disturbed and short term reactions are applied.

The particular factor of interest addressed here, is the applied planning horizon of a human. This aspect specifies how far into the future a human plans its motion. For locomotion, this comprises how far a human looks ahead, to what extent he predicts other agents’ motions, and whether he plans the full trajectory to the goal or only a few steps ahead. Our hypothesis is that humans resolve situations such as near-collisions, unanticipated occurrences or cases of failed prediction by adapting their planning horizon. This behavior is necessary, since these situations are the result of the human’s uncertainty about the exact motions of all other agents within the current environment. We summarize these uncertain situations as “partially observable environments” and assume that our hypothesis generalizes to most of this collectivity [13]. Within this work, sufficiently complex environments are also assumed as partially observable, because a human being is not able to track more than a certain number of agents at once [13]. In contrast to the posed hypothesis, a human is expected to follow the unique optimal solution, if the environment is “fully observable” and thereby carries no uncertainties.

Within recent literature, humans are usually considered to plan an optimal trajectory from their current position to a defined goal. The applied planning horizon covers the whole trajectory, while factors like time, path length and energy expenditure are optimized [7, 8, 14]. However, these methods are not able to reproduce the exact strategy employed by humans, if unpredicted disturbances occur [4, 10, 15]. Therefore, different approaches are found in literature that are applicable as corrective measures: constant re-planning [16, 17], re-planning at specific states [18, 19], integration of intermediate goals [4], or reactive approaches without prior planning [20]. With these approaches, the observed trajectories are replicable but the underlying human locomotion behavior remains unclear. A detailed model for the human behavior to handle disturbances would thus allow for more accurate predictions. In order to tackle this problem, we must explore whether humans employ different behaviors within fully observable and partially observable environments. In this work, we follow the idea that humans employ a shorter planning horizon in complex or uncertain scenarios [16]. Thereby, it is not known how much complexity humans can handle before they start to adapt their planning. A second issue is the fact that the executed trajectory may diverge from an optimal solution and lead to a suboptimal motion [10]. Therefore, it is also of interest if the executed trajectories stay within certain boundaries around the initial optimal solution. The resulting goal is a specialized experiment, which aims to determine if a shorter planning horizon constitutes a specific human re-planning strategy.

Throughout this work, human locomotion prediction is approached from a control perspective, assuming a human as a dynamic system that optimizes its locomotion with respect to aspects like energy consumption. Based on existing models [4, 8], the prediction problem is formulated within a nonlinear model predictive control (NMPC) framework. The influence of the planning horizon is then initially analyzed within simulations. This framework and the respective simulation results allow us to illustrate the problem regarding the human planning horizon from a control theoretic point of view. Detailed statements about this human behavior, however, require further evaluations in user studies.

In order to investigate the mentioned aspect, an experiment is devised and conducted, which aims to yield basic insights into the human motion planning process. Indeed, measuring the currently applied planning horizon of a human is challenging, as it is the product of cognitive elements which currently cannot be detected directly by sensor-based approaches. The complexity of this process is illustrated by Goffman’s theory about interactive human locomotion behavior [21]. On this basis the “sense-plan-act” architecture [22] is established as a cognitive model. It stipulates that pedestrians attempt to sense where other humans intend to go and then adapt their own plans to move accordingly. This cognitive model comprises the idea of a planning horizon which starts with sensing and ends with the action. Thus, three distinct tasks define this process: perception of the environment, planning of a path taking into account the predictions of all agents and execution of the trajectory. This point of view also establishes an association between the planning horizon and the visual look-ahead. Clearly, planning further into the future requires humans to gather more visual information and to extend their predictions. In order to measure this process empirically, the start- and end-points are of main interest. Therefore, we propose an experimental setup that measures the visual focus and the trajectories of subjects that perform movements within a specifically designed virtual environment. The subjects’ movements are additionally disturbed by other virtual objects to trigger the anticipated avoidance behaviors. Changing the observability and therefore raising the uncertainty is accomplished by altering the complexity of the virtual environment based on the number of obstacles [16]. This experimental design is expected to reveal information about the planning horizon applied by humans and the accepted deviation of trajectories from distinct optimality criteria.

In summary, this work features empirical research, which investigates adaptations in the human motion planning horizon in order to enhance human locomotion prediction. Based on theories regarding the cognitive process of trajectory formation, an experimental setup is proposed to verify the hypothesized behavior. The experiment further constitutes a foundation for future investigation of this aspect, since the planning horizon was only marginally considered in previous research. Analyzing how humans adapt their planning horizon, yields the opportunity to improve optimal control based motion prediction methods by incorporating the identified behavior. Especially the prediction of specific avoidance and recovery motions, which emerge from reactions to high collision risk, is expected to be improved. In addition, it is investigated whether humans deviate from an optimal solution during an avoidance motion and, if so, to which extent. Thereby, the presumed behavior is a confinement to a convex hull which forms a corridor between the current and the goal location. This would further indicate that complex scenarios are handled with a shorter planning horizon.

Clearly, dynamic environments require a human to re-plan and adapt its locomotion trajectory in case of disturbances. Therefore, knowledge of the way humans adapt their planning horizon will allow robots to predict human motion more accurately in complex situations. Since the planning horizon seems to be strongly connected to collision avoidance behaviors, the varying results towards velocity and path adaptation shown in literature may find more explanation within our work [10, 11]. The results of the proposed study and possible continuations potentially influence future motion prediction approaches, such that a wider variety of human behaviors is accurately represented. This advance can influence the collaboration with robots during locomotion as well as collaborative manipulation. The accuracy of predictions strongly determines the seamless integration of robots in a shared workspace and eventually the efficiency of this collaboration. Robots that move along with humans in warehouses or provide them with tools or parts during assembly must become convenient and efficient collaboration partners and therefore support as well as utilize mutual predictions.

Subsequent work has the following structure: In the Related Work section literature regarding motion prediction and avoidance behaviors is discussed. The Problem Description elaborates the applied cognitive model and formalizes an NMPC framework accordingly. The following section regarding the Empirical Exploration of the Human Planning Horizon describes the design and procedure of the conducted experiment. Obtained results are presented in the Main Experiment Results section. After a short summary, the experiment is discussed and conclusions are drawn in the Discussion and Conclusions section.

## Related Work

The following section discusses literature which is of relevance to this work. Firstly, the minimum effort principle is presented, which is a fundamental pattern within human locomotion. This is followed by a short overview of common motion prediction approaches that integrate human behaviors. Thereafter, prediction of human locomotion trajectories based on optimal control is discussed. Correspondingly, works on predicting avoidance behaviors are discussed, which are based on optimal control models as well. Specific weaknesses within these respective models are revealed and their relation to the planning horizon are highlighted. In opposition to the optimal control based models, literature on behavioral locomotion models is briefly discussed. Furthermore, cognitive models are accounted for, which support the paradigm of an adaptive planning horizon in human locomotion. Lastly, literature on the relation of the planning horizon to the behavior of looking ahead is briefly depicted.

Motion prediction is a wide field applicable to any mobile agent, e.g. humans, cars or robots. The methods are widespread and usually generalize to a large variety of situations. A fundamental fact that influences many prediction algorithms is that humans intend to walk with minimum effort regarding energy and cognitive strain [23–25]. In a fully observable environment, humans are able to follow this principle successfully. Accordingly, effort is minimal since the initial locomotion plan is not disturbed. On the contrary, to cope with partially observable environments, re-planning and trajectory adaptations are necessary, which cost energy and lead to cognitive load. This is not desirable for a human but certain situations require this flexibility in order to circumvent dynamic obstacles successfully. This correlation strongly influences our work as it yields a measure for seamlessness and a feature which marks the desired undisturbed motions. Accordingly, high efforts due to uncertain situations or disturbances from surrounding agents are to be avoided by modeling human trajectory planning more accurately and by improving the prediction of humans. The mentioned feature is used in our analysis and allows us to interpret whether a subject has used an undisturbed trajectory or had to re-plan.

A survey on recent prediction methods applied to autonomous cars is found in [3]. Many prediction algorithms are based on Kalman filtering [5, 26, 27], which does not yield good performance for complex environments. Multiple hypotheses are fused with a Kalman filter in [28], to predict future positions of humans. Here, social aspects are considered to play a crucial role in avoidance and prediction. A very influential work towards prediction of pedestrian locomotion is proposed by Ziebart et al. [6]. Inverse optimal control is applied on top of a Markov decision process to learn the preferred paths of pedestrians with respect to the environment. The approach enables a robot to position itself in a least interfering way or plan its path according to this measure. Incorporation of human behaviors is implicit and generalization to arbitrary environments is possible. Yet, for applications where the accurate trajectory as well as accelerations and torques are necessary, dynamic model based approaches are advantageous. The work of Kuderer et al. [29] is methodologically similar to [6] as it proposes to learn features of the environment in a similar way. Indeed, this work also shows the importance of continuous trajectories and the consideration of velocities as well as accelerations. In accordance to that, the work at hand is concerned with accurate prediction methods based on dynamic models and optimal control theory.

Accurate prediction of human locomotion trajectories using optimal control and a unicycle model is widely studied [4, 7, 8, 30, 31]. These works propose different objective functions that reduce the solution space to a subset that closely resembles human locomotion trajectories. Thereby, some works focus on minimization of energy, path length and time, whereas others follow specific curvature constraints. In fact, most approaches are developed with the goal of locomotion prediction. An inverse optimal control approach is shown by Mombaur et al. [8]. The method allows to incorporate new objective functions into a holonomic model and estimates their influence. In [14, 32], prediction for arm movements based on the same methodology is presented. These approaches also opt to generate human-like motions and investigate the underlying objectives. In [9] the generation of human locomotion paths is addressed similarly to aforementioned works. Here, the problem is reduced to the path data in order to gain invariance to velocity, although other works consider both aspects to be strongly correlated. The authors reformulate the problem from a constrained into a convex unconstrained least-squares optimization. An adapted inverse optimal control approach is applied that incorporates the discrete Frechét distance and leads to new cost functionals for human locomotion. A comparative evaluation is shown in [33]. In [34] trajectory prediction methods based on optimal control and spline fitting techniques are compared. Multiple predictions between a current position and all estimated goals are taken into account. Selection of the most likely trajectory is done using minimum curvature variation, path length and execution time. In [35] authors propose that humans plan full trajectories to a goal rather than a series of steps. Subjects varied their foot placement within repetitions of the same path, suggesting that goal-oriented locomotion is related to higher level trajectory planning rather than step planning. Humans are also considered as optimal controllers in [36]. Here, the optimal control approach is used to predict pedestrian behavior in order to improve building layouts. Bascetta et al. [26] combine the optimal control procedure with Kalman and particle filters. This enables short term prediction for a human-aware robot cell but only for a single human.

All mentioned approaches consider humans as optimal controllers and aim to identify the composition of objective functions, which are used to predict human locomotion. For this prediction it is assumed that humans always plan trajectories between their current position and a defined goal. Our work adopts this methodology but directs the attention to the inherent aspect of the real planning horizon, which is barely addressed. The results of the work at hand will provide insights into human behaviors which could improve the precision of existing prediction models and methods.

Avoidance behaviors of humans are often investigated in user studies where an interfering but not interacting person (often called intruder) crosses the subjects path from the side [4, 10, 11, 37–39]. This case is studied intensively based on optimal control models which are applicable to robot navigation. Interest is particularly set on avoidance strategies employed by the human being. Authors repeatedly report either velocity or path adaptations as the reasons for observed trajectories, but a common principle is not defined. Some approaches assume velocity adaptations as the typical behavior [10], whereas others propose the combination with path adaptation as the underlying principle [39]. Rule based behaviors following time-to-collision or minimal-predicted-distance [18, 19], pose another method to model the timing of avoidance movements. These features model the re-planning at specific positions relative to the obstacle. The most influential idea to our work is followed by [4], which applies an adaptation to its initial methodology. Albrecht et al. integrate obstacles into their framework which makes it well applicable to trajectory prediction problems and to predict avoidance behaviors. Indeed, the authors successfully predict a trajectory of a free-space walk, but need to add a re-planning structure based on a distance rule to approximate human data that contains disturbances from an interfering agent. Clearly, the human data shows a behavior that strongly diverges from the optimal control idea of full observability and a fixed control horizon. The approach to replan and thus adapt the optimal trajectory leads us to the question whether humans behave in a similar way and adapt their planning horizon.

The inaccuracy of current optimal control models and the disagreement between different avoidance studies arises from missing knowledge about underlying parameters and behaviors. Especially the inaccuracies reported in [4, 10] have not been addressed, yet. We assume that the planning horizon is one important parameter in this regard. Hence, the results of this work yield valuable ideas and insights to clarify this divergence. In order to investigate this factor on a model based level, the work of Albrecht et al. [4] is used and reimplemented using the ACADO Toolkit [40, 41].

In contrast to the optimal planning procedures, some works analyzed the reactions of humans during locomotion and provide behavioral models to describe the short term behaviors. These approaches often consider humans as simple controllers that change their behavior based on a set of rules. Obstacle avoidance is thereby based on distances rather than a prediction horizon. This rule based behavior resembles a very short planning horizon in contrast to the mentioned optimal control methods. Some related publications are presented in the following. A fundamental work in the field of human locomotion behavior is provided by [20]. Fajen et al. propose a human inspired constant velocity steering model. Their experiments validate the approach for static environments where obstacles may suddenly appear. Fink et al. [42] evaluate the difference of locomotion paths in real and virtual environments. The results show that humans are able to project their physical behavior. This supports the use of virtual environments for experiments regarding human motion. In [43] a velocity based model for the locomotion behavior of a human crowd is proposed. This model employs the principles of personal space, least effort and time-to-collision. Using a fixed horizon for taking obstacles into account is also proposed in [44]. In relation to a planning horizon, obstacles are considered only within a certain distance and independent of the individual speed. In [45], aspects that influence the use of an open-loop or closed-loop methodology for locomotion control are investigated. This matter is specifically investigated for the absence of visual feedback and for varying velocities. Pham et al. attempt to clarify whether humans use a feedback scheme for locomotion, given they act like a controller. The consideration of closed-loop and open-loop structures shows many parallels to our work.

Behavioral models where humans react to obstacles based on a fixed set of rules, resemble a very short planning horizon. Planning ahead in order to find an optimal solution, as it is the case for optimal control methods, is not considered. The results of these methods show that human locomotion behavior is also predictable with a very short horizon. Yet, optimal control methods yield a better performance when optimal solutions are required to reproduce human behavior in comparison to a forward simulation of the short-term behaviors.

Human locomotion planning is a complex cognitive process, which is investigated in this work. Fundamental literature that describes the cognitive processes considered in this work is posed by Goffman [21] and Reich [15]. Goffman describes the human locomotion behavior as a series of actions. Humans “externalize” their intention (e.g. their goal) nonverbally by stereotypical movements, gaze or heading. Then they try to “sense” what others intend and incorporate this into their own planning process. Finally, under consideration of sensed information and own intentions a trajectory is executed. This sequence is repeated constantly in order to avoid collisions and reach the personal goal. This loop is termed “sense-plan-act” in [15], resembling a typical cognitive control loop used in robotics [22]. A faster model is proposed as “sense-control-act” that reduces the trajectory planning to an adaptive steering to the intended goal position. Apart from that, [15] also poses the question for the correct timing to initiate re-planning. The present work yields results towards an answer. The authors of [16] propose to model the cognitive path planning process in the human brain with an MPC approach. They hypothesize that the cognitive load rises with more complex and longer paths to be planned.

The two cognitive loops proposed in literature obviously postulate a long (start to goal) and a short (reactive) planning horizon. The “sense-plan-act” idea favors the planning of longer progression periods, which could be whole trajectories, while “sense-control-act” reduces the planning to a short term reactive behavior. Similar to the different approaches of modeling human locomotion behaviors, this disagreement supports our assumption that this matter is not unanimously defined.

Perception plays a central role during motion planning and is also a key action in the presented cognitive models. It is thus considered as an indicator for the planning horizon in our work. Some experiments in literature are concerned with the visual look-ahead that humans employ during navigation tasks. Look-ahead during steering around obstacles on a bike in a virtual environment is considered in [46]. Authors analyze how the fixation of near and far obstacles develops during the course and find that fixation occurs with regard to the closest obstacle and switches to the next obstacle at a distinct distance. Look-ahead during foot placement on a predefined parcour is the topic of [47]. Results point towards interesting behaviors when the final pose is approached. The influence of the planning horizon of a robot on its apperception is addressed in [37]. It is shown how constant re-planning leads to undesired behavior if the environment is not fully observable or the planning horizon is too short. Clearly, findings about look-ahead behavior relate to the planning horizon addressed here. However, apart from [45], a change in the planning horizon has not yet been investigated directly or considered as a critical factor in human behavior.

Literature in the area of robotics, optimal control, experimental psychology and clinical research has not directly evaluated the applied planning horizon within human locomotion planning as an influential parameter for prediction. The idea of a change in the hypothesized planning horizon is also not further investigated in the literature regarding control theoretical models. Therefore, the investigation of the applied planning horizon can contribute towards identifying the set of behaviors that are necessary for accurate human locomotion prediction.

## Problem Description

In the following, a model for the planning horizon applied by humans during locomotion is defined. The framework comprises a cognitive process suggested in literature and a control theoretic structure which defines the planning behavior in more detail. Simulations of the planning architecture allow for basic insights into the effects of a change to the applied planning horizon. The results will further motivate the investigation of this aspect.

### Cognitive Architecture for Human Locomotion

Human behavior during locomotion is described as a repetitive process consisting of: gathering visual information [47], constructing a trajectory to the goal [35] and executing this trajectory. Planning and acting are thereby strongly affected by the human ambition for minimum effort [24]. A descriptive cognitive process, which underlies human motion, is supplied by Goffman [21] and Reich [15]. The cycle is summarized as “sense-plan-act” in [15] and already found application in the early years of robotics [22]. Reich [15] builds upon this model and proposes a “faster” loop: “sense-control-act”. This structure is cognitively less demanding and allows a human to walk towards its goal while steering around obstacles without the planning or re-planning of a detailed path. A realization of this shorter cycle is described within [20] in the Related Work section. Both cognitive models are applicable to reproduce human locomotion paths, but the actual human planning horizon and whether this horizon can change, is not yet clarified.

Investigating the human planning horizon requires the measurement of automatic processes that occur in a subject’s mind. However, this cognitive loop is neither directly observable for sensors nor derivable from questionnaires. The described models are therefore an indication of how an experimental setup is able to measure the process. Only input and output information are measurable entities. Following the models, the input consists of visual information and the output is posed by the traversed trajectory.

Accordingly, an experiment needs to be designed that asks a subject to plan and execute a motion in a visually observable environment. Further, in order to trigger changes in the planning horizon, the experiment needs to provide fully and partially observable situations. As mentioned before, the complexity and therefore the observability of a virtual environment is easily adapted by changing the number of obstacles. Thus, the development of the gaze and trajectory data is evaluated, while subjects are presented with various situations. The expectation for simple unobstructed environments is to observe a smooth and immediate movement from a defined start to a defined goal location. Measured sensing data, i.e. gaze, is expected to focus on the goal mostly. On the contrary, a jerky motion is expected for partially observable environments, with stepwise movements (acceleration and braking) and delimited looking ahead. Following this, we expect that the human planning horizon is proportional to the applied visual anticipation of the person. If the extent of visual anticipation is a precondition for the planning horizon, a correlation to the smoothness of executed motions must be visible. Given that the result of a measurement is a two dimensional planar trajectory *ξ* = [(*p*_*x*_(*t*), *p*_*y*_(*t*))|*t* = 0, …, *T*], which describes a change of position over time, between a start point (*p*_*x*_(0), *p*_*y*_(0)) and an end position (*p*_*x*_(*T*), *p*_*y*_(*T*)). The trajectory *ξ* and especially the velocity profile $v\left(t\right)=\dot{\xi }$, are smooth between *ξ*(0) and *ξ*(*T*), if the obstacles are well predictable for the subject. This implies that the trajectory smoothness is proportional to the planning horizon and the visual look-ahead. Given a complex scenario, smoothness is expected to be maintained between an arbitrary starting point and a position close to a predicted obstacle trajectory. It is assumed that smoothness diminishes to a concatenation of movements for more complex environments. Generally, complex situations pose a high level of uncertainty for a human agent. Therefore, the applied planning horizon is assumed to depend on the uncertainty about an environment.

Overall, the cognitive models and literature about human locomotion lead to a set of proportions which are measurable during an experiment: visual fixation, smoothness of velocity and path and the complexity of an environment, which we assume to correlate with observability and uncertainty [16]. From these factors, adaptations of the planning horizon of a human subject are deduced. The specific methods for measuring these aspects are explained in detail in the section regarding the Empirical Exploration of the Human Planning Horizon.

### Planning Horizon in NMPC Locomotion Prediction

**Fig 1:**
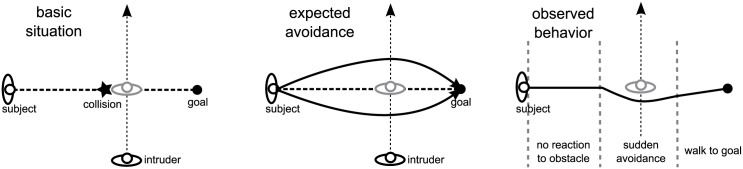
Illustration of an observed deviation from the typical avoidance behavior of humans.

In this section, the influence of the planning horizon in human locomotion prediction is elaborated. The basic problem is posed by a moving human which is disturbed in his progression by another agent in the same environment. Optimal control methods usually yield accurate prediction results for this situation, if the interfering agent is well predictable. These methods assume a planning horizon that spans the whole trajectory between a start **p**_*S*_(0) and an end position **p**_*G*_(*T*). Optimal control thereby follows the theory that humans intend to walk with minimum effort [23]. In a fully observable environment, where the trajectories of all agents are reliably predicted, humans are able to follow this principle. Thus, optimal control methods will produce reliable predictions. However, many experiments in literature show that humans tend to deviate from this minimum effort behavior [4, 10, 11]. Predictions with optimal control methods are shown to be unable to reproduce the trajectories observed in these cases. Hence, human locomotion appears to follow different suppositions for some situations. Optimal control based prediction approaches do not generalize to these changes in behavior and therefore need to be improved. Fig 1 illustrates the results of these previous investigations. In order to obtain a suitable prediction for this case, the optimal control structure is changed to an MPC structure in [4]. This approach basically re-plans the trajectory based on a simple distance rule. It appears that humans do not necessarily consider all obstacles from the beginning and apply a shorter planning horizon than the optimal control methods assume.

Given that even a single obstacle leads to sudden avoidance behaviors, it can be assumed that humans change their behavior if they are confronted with uncertainties. For example, unreliable predictions for other agents or high complexity of the environment (e.g. many obstacles) lead to large uncertainties for a human. Surprising behavior of an agent, like a sudden change of direction, may have equal effects. These situations require the flexibility from humans to omit their minimum effort strategy. Our work summarizes situations with high uncertainty as partially observable environments and proposes that humans alter their planning horizon to cope with them. The subsequent simulations investigate this proposition and show that a shorter planning horizon reproduces the observed behavior in [4].

**Fig 2:**
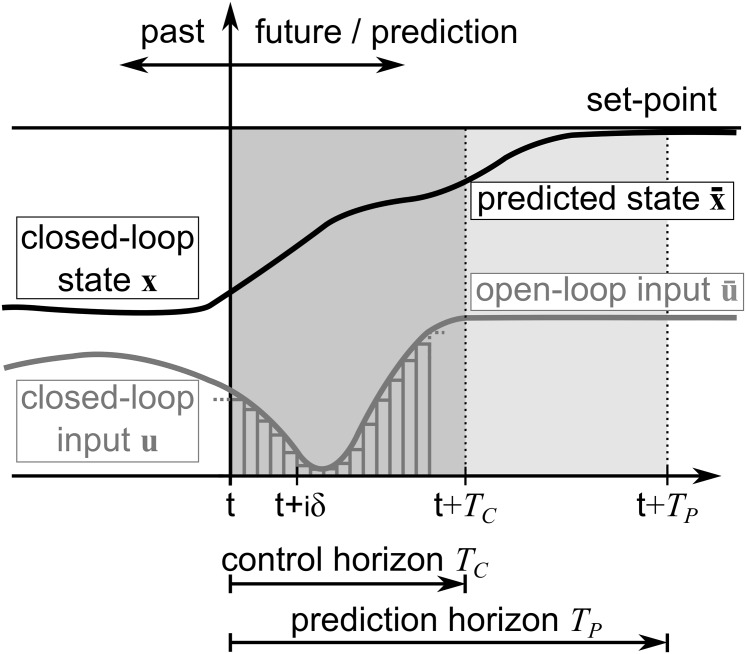
Illustration of the NMPC process.

In order to illustrate the problem in more detail, humans are modeled using NMPC, as it is proposed in [4, 35]. This allows to analyze effects of the adaptation of the planning horizon within the modeled human locomotion behavior. Notation from [41] and simulations with the ACADO Toolkit [40] are used to recapitulate the problem. The NMPC model gives an estimation of the trajectory for the considered situation. As the controls are not applied to a system, the simulation results resemble a model based prediction rather than the controlling of a human. For prediction of human locomotion, control and prediction horizon have equal length *T*_*C*_ = *T*_*P*_. This originates from the incorporation of a goal pose which is necessary to constrain the infinitely possible motions to a reasonable set. Based on that, a two-point boundary value problem is solved directly at each time-step *δ*. The initial boundary is formed by the current closed-loop states **x**(*t*) and the closed-loop controls **u**(*t*) at the starting time *t*. As a final boundary the goal pose **p**_*G*_(*T*) = (*x*_*G*_(*T*), *y*_*G*_(*T*), *φ*_*G*_(*T*)) is used which also serves as set-point. Solving the problem yields state predictions $\bar{\text{x}}\left(t\right)$ and the inputs $\bar{\text{u}}\left(t\right)$. Fig 2 illustrates the NMPC process.

As already proposed in [4, 7, 8], the dynamic model $\dot{\text{x}}\left(t\right)=f\left(\text{x}\left(t\right),\text{u}\left(t\right)\right)$ with **x**(0) = *x*_0_, resembles a unicycle:

$$\begin{array}{c} \dot{\text{x}}\left(t\right):=\frac{d}{dt}(\begin{array}{c} p_{x}\left(t\right)\\ p_{y}\left(t\right)\\ \varphi (t)\\ v(t)\\ \omega (t)\\ a_{v}\left(t\right)\\ a_{\varphi }\left(t\right) \end{array})=(\begin{array}{c} v(t)\cos(\varphi (t))\\ v(t)\sin(\varphi (t))\\ \omega (t)\\ a_{v}\left(t\right)\\ a_{\varphi }\left(t\right)\\ u_{1}\left(t\right)\\ u_{2}\left(t\right) \end{array}), \end{array}$$

with constrained states and inputs:

$$\begin{array}{ccc} \text{x}(t) & \in & X,\forall t\geq 0,\\ \text{u}(t) & \in & U,\forall t\geq 0, \end{array}$$

where $\text{x}\left(t\right)\in R^{n}$ and $\text{u}\left(t\right)\in R^{m}$. Following [41], the sets X and U are compact, e.g. box constraints:

$$\begin{array}{ccc} X & := & \{\text{x}\in R^{n}|{\text{x}}_{\text{min}}\leq \text{x}\leq {\text{x}}_{\text{max}}\},\\ U & := & \{\text{u}\in R^{m}|{\text{u}}_{\text{min}}\leq \text{u}\leq {\text{u}}_{\text{max}}\}, \end{array}$$

where **x**_min_,**x**_max_,**u**_min_,**u**_max_ are constant vectors. Within NMPC, an optimal control problem with finite horizon is solved repeatedly by optimizing an objective functional *J* with runtime cost *ϕ* weighted by *θ*_*i*_:

$$\begin{array}{c} \underset{\bar{\text{u}}\left(\cdot \right)}{\text{argmin}}\;\;\;J\left(\text{x}\left(t\right),\bar{\text{u}}\left(\cdot \right);T_{C},T_{P}\right), \end{array}$$

with

$$\begin{array}{c} J\left(\text{x}\left(t\right),\bar{\text{u}}\left(\cdot \right);T_{C},T_{P}\right):=\theta_{i}\int_{t}^{t+T_{P}}\phi (\bar{\text{x}}\left(\tau \right),\bar{\text{u}}\left(\tau \right))d\tau , \end{array}$$

subject to equality constraints:

$$\begin{array}{ccc} \dot{\bar{\text{x}}}\left(\tau \right) & = & f\left(\bar{\text{x}}\left(\tau \right),\bar{\text{u}}\left(\tau \right)\right)\;\text{with}\;\bar{\text{x}}\left(t\right)=\text{x}\left(t\right),\\ \bar{\text{u}}\left(\tau \right) & = & \bar{\text{u}}\left(\tau +T_{C}\right),\;\forall \tau \in \left[t+T_{C},t+T_{P}\right],\\ T_{P} & & \;\text{free}, \end{array}$$

inequality constraints:

$$\begin{array}{ccc} \bar{\text{u}} & \in & U,\;\forall \tau \in [t,t+T_{C}],\\ \bar{\text{x}} & \in & X,\;\forall \tau \in [t,t+T_{P}], \end{array}$$

and boundary conditions:

$$\begin{array}{c} b(\bar{\text{x}}\left(t\right),\bar{\text{x}}\left(t+T_{C}\right),\bar{\text{u}}\left(t\right),\bar{\text{u}}\left(t+T_{C}\right))=0. \end{array}$$

Iteratively solving this optimization problem results in the open-loop solution for the problem ${\bar{\text{u}}}^{*}\left(\cdot ,\text{x}\left(t\right),T_{P}\right):\left[t,t+T_{P}\right]\to U$. The optimal solution for the closed-loop system is a sequence of open-loop solutions:

$$\begin{array}{c} {\text{u}}^{*}\left(\tau \right):=\bar{\text{u}}\left(\tau ,\text{x}\left(t\right)\right)\;\text{with}\;\tau \in \left[t,\delta \right]. \end{array}$$

The “nominal closed-loop system” is:

$$\begin{array}{c} \dot{\text{x}}\left(t\right)=f\left(\text{x}\left(t\right),{\text{u}}^{*}\left(t\right)\right) \end{array}$$

For the direct solution a finite parametrization of the controls is used, which leads to a finite dimensional dynamic optimization problem. Therefore, the controls are constant over each sampling interval $M=\frac{T_{P}}{\delta }$ such that $\bar{\text{u}}\left(\tau \right)={\bar{\text{u}}}_{i}$ for *τ* ∈ [*τ*_*i*_, *τ*_*i*+1_) with *τ*_*i*_ = *t* + *iδ*. Applying a “sequential approach” [41], the control vector ${\bar{\text{u}}}_{i}=\left\{{\bar{\text{u}}}_{1},\cdots ,{\bar{\text{u}}}_{M}\right\}$ is optimized, resulting in the optimization problem:

$$\begin{array}{c} \underset{{\bar{\text{u}}}_{i}}{\text{argmin}}\;\;\;J\left(\text{x}\left(t\right),{\bar{\text{u}}}_{i},T_{P}\right) \end{array}$$

where only **x**(*t*), the input vector ${\bar{\text{u}}}_{i}$ and *T*_*P*_ = *t* + *Mδ* appear. With this NMPC framework and the objective function as well as the constraints from [4], simulation results are obtained that highlight the influence of the planning horizon. We compare an optimal control (OC) solution with the NMPC solution to determine which method replicates the behavior shown in literature best.

**Fig 3:**
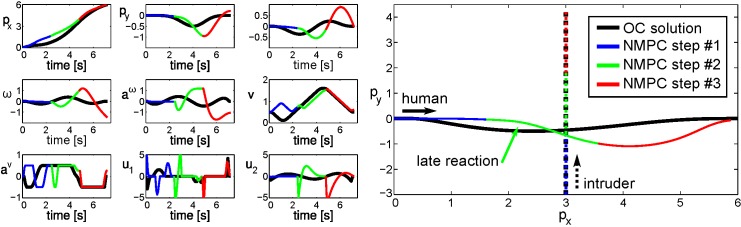
OC solution (black) compared to NMPC solution with the shorter planning horizon (three steps colored). States and controls are subject to time (*t*). The path of the intruder that disturbs the motion is depicted as a dashed line. The lengths of the lines match the time horizons.

The OC method uses a time horizon of 7.2*s* which covers the whole trajectory from the start to the end. For the NMPC the planning horizon is set to *T*_*P*_ = *T*_*C*_ = 2.4*s*. The set-point at **p**_*G*_(*T*) = (6.0, 0.0) is necessary to constrain the solution space of the unicycle model to a reasonable set. Most constraints in the objective function of [4] also depend on this final pose. Thus, it is assumed that the set-point is known, whereas in a prediction scenario this is not the case. Omitting the final pose in order to generate a more generalizable OC based prediction method is not in the focus of this work. Fig 3 illustrates the comparison of the methods. We consider the scenario where an intruder is crossing the human’s path at 90 degree and interferes with its intention to walk straight to the goal. The intruder starts its slow walk with 1.0m/s at a position (3.0, −3.0), which is also three meters away from the crossing point of the straight paths of both agents. A subject has two options in this case, to pass behind or in front of the intruder. With the desired velocity of the human set to 1.4m/s, see [4], the OC and the NMPC solution let the interfering agent pass first. The black path in Fig 3 is the result of the OC method, which considers a fully observable environment. Clearly, the avoidance maneuver is initiated right from the beginning, because the future positions of the interferer are known. Controls, velocities and states progress smoothly without extensive energy expenditure. The velocity *v*(*t*) reveals, that the OC solution brakes and accelerates to let the intruder pass. For comparison, the colored paths show the NMPC result. The obstacle is not considered in the first part (blue) because the planning horizon is reduced. Within the second part (green), the planning horizon reaches the obstacle and a reaction is initiated. State and control plots reveal, that this reaction requires far higher energy expenditure than the OC solution. The velocity plot also indicates the braking, but a smooth progression is not achieved due to the reconsideration of the problem after the first prediction horizon. The last part of the NMPC result (red) predicts a swerve back to the set-point, which shows smooth states and controls due to the free path. Necessary controls during the shorter planning horizon illustrate why the OC solution is usually preferred. Yet, these results appear very similar to the observations of related literature, as illustrated in Fig 1.

The differences in the paths from these simulated situations match the statements of [4, 10], that humans do not strictly follow the OC idea. Within mentioned work, the OC approach shows inaccurate predictions. It is further shown, that humans seem to resolve collision situations with a shorter planning horizon. Presented simulations support this observation. We follow the idea that humans walk smoothly from start to goal if no information is hidden for them. In case of unexpected events, unreliable predictions or other uncertainties, we assume that humans employ quick adaptations which lead to suboptimal and jerky recovery motions. OC is able to produce a prediction for the optimal trajectory, whereas the NMPC solution is not necessarily optimal but resembles the solution for a shorter planning horizon. NMPC is further capable of attuning to model inaccuracies and changing environments, e.g. when a dynamic obstacle suddenly stops or its prediction is inaccurate.

Viewing human locomotion as a NMPC framework, allows us to correlate the human planning horizon with a methodology. As the simulations illustrate, changes of this aspect with respect to environment observability, lead to very different results. Considering the influence of the planning horizon on prediction accuracy, it appears beneficial to investigate this aspect. Accordingly, the properties of the planning horizon that humans employ during locomotion are explored within subsequent experiments.

## Empirical Exploration of the Human Planning Horizon

This section describes the experimental design which is developed to tackle the difficulty of measuring the planning horizon employed by humans. The experiment is designed as a study with human subjects that perform a goal-directed motion. As mentioned before, measuring a cognitive process is not achievable using sensors. An experiment needs to visualize the aspects that are associated to the process, to eventually allow for conclusions about the underlying planning behavior.

### Experiment Design

At first, a setup is needed where a subject is required to perform a goal directed motion. A large room equipped with a tracking system or a virtual environment are suitable setups, as they allow to measure the motions. In order to measure changes in the applied planning horizon, the experiment must feature multiple comparable conditions. Thus, a subject will perform the goal directed motion multiple times in varying environments. Generally, the environments will differ in the number of moving obstacles that need to be passed without colliding. Therefore, stable environment conditions must be provided for all subjects to support comparable and unbiased data. This includes that interfering obstacles move equally for all repetitions of the experiment. Within a motion capture area, where subjects walk freely to their goal, moving obstacles are representable by interfering human agents. However, providing consistent conditions for all subjects is exceedingly difficult to achieve in such a setup. A solution would be to use multiple robots or other controllable hardware which behaves in exactly the same manner towards all subjects; but apart from the excessive effort and cost, safety for the subjects is a major concern with this approach. Therefore, we consider virtual environments as they offer measurability and control of all parameters as well as flexibly adjustable complexity. Walking in virtual reality unfortunately requires hardware that was not commonly accessible when the experiments were conducted. Hence, an alternative is needed that allows subjects to perform a goal directed motion in a natural and intuitive way. Since the planning of arm motions and locomotion show comparable aspects and are based on similar control theoretic foundations [8, 14], arm motions were considered as a substitute. In literature, the direct comparison of both motion types reveals clear similarities [48, 49]. It was also shown that locomotion and arm motions are controlled by the same region of the brain [50]. Models developed to describe arm or hand movements are also successfully applied to model locomotion [32, 35]. For this substitutional setup a kinesthetic device with a virtual representation of the motion was available [51]. The “Desktop Kinesthetic Feedback Device” (DeKiFeD) [51] is a four degree of freedom (DoF) interface. By using this device, subjects perform a natural and intuitive motion with their arm to follow a trajectory which they have planned. The “sense–plan–act” model is triggered as subjects see their progress as well as obstacles in the virtual representation. This enables us to ask subjects to perform goal-directed motions in an observable and fully controllable environment. Since it was not clear whether the experiment yields data to support the hypothesis of a changing planning horizon, we choose this setup as it poses an appropriate trade off between installation effort and expected outcome.

#### Measuring Parameters of the Planning Horizon

With subjects performing motions in a virtual environment, we are able to provide them with different conditions to elicit changes in their planning horizon. As it is not possible to directly measure the planning horizon, other parameters must be observed to gather interpretable information. As stated in [16], with an increasing complexity of the environment (e.g. dynamic obstacles), cognitive load for locomotion planning will rise and a shorter planning horizon is employed. However, measuring the cognitive load is not sufficient within the proposed study setup, as the increase in cognitive load due to planning may be superimposed by the effort for steering and observing the environment. This issue is tackled by recording other parameters which resemble the planning horizon. With respect to the overall design, gaze tracking offers a direct measurement of the “sense” input to the cognitive process. Under the assumption that the planned trajectory is restricted to the observed part of the environment, a human is expected to look ahead less far if the planning horizon is short. In addition, measuring path and velocity data yields further information about the smoothness of the planned motion. We assume that subjects avoid one or two moving obstacles easily, while maintaining smooth progression. However, the smoothness of both path and velocity should diminish with a shorter planning horizon in more complex scenarios.

#### Triggering Adaptations of the Planning Horizon

In order to investigate the planning horizon, the experimental conditions must demand a subject to gradually decrease the planning horizon. With respect to the Problem Description section, this decrease must be triggered by increasing the uncertainty for the subject. As mentioned, higher uncertainty is achieved by rendering the environment partially observable due to a rising complexity of the situation. For the virtual environment, this is realized by increasing the number of moving obstacles. A subject is able to track and predict a few obstacles in its way, but with increasing numbers the uncertainty will rise. This way, the gradual influence is realizable and different situations are comparable.

We assume that subjects attempt to solve the posed motion problem optimally in case of a simple and therefore fully observable environment. This allows for the comparison whether subjects omit the globally optimal solution when a shorter planning horizon is necessary. An optimal solution is defined by the subject’s tasks. The task description defines that the goal position must be reached with least possible collisions, whereas the elapsed time is not of any meaning and the collisions are not counted. In order to solve the posed motion problem optimally, a simple straight motion from the start to the goal position is sufficient. Indeed, the study setup provides an optimal solution in every condition, which allows to move to the goal by a single smooth motion. Owing to the structure of the virtual environment the opportunity for this optimal path appears periodically, but specifically two seconds after the start of a trial. In correspondence to [52] a human is capable of observing and assessing the environment in less than two seconds. But the subject must be capable of observing and predicting all obstacles in order to find this solution. Note, that owing to the supplied top view, subjects see all obstacles at every time step of their motion planning. Yet, a subject may also move to the goal on a straight line while avoiding collisions step by step.

The hypotheses about the planning horizon and the deviations from an optimal solution are now investigated by measuring the paths, velocities and major gaze fixation areas of each subject. Rising numbers of obstacles are supposed to result in partial observability and a reduction in the planning horizon. Accordingly, fluctuating velocities, lower average look-ahead and deviations from the theoretically optimal path are the expected consequences.

#### Setup of a Virtual Environment

**Fig 4:**
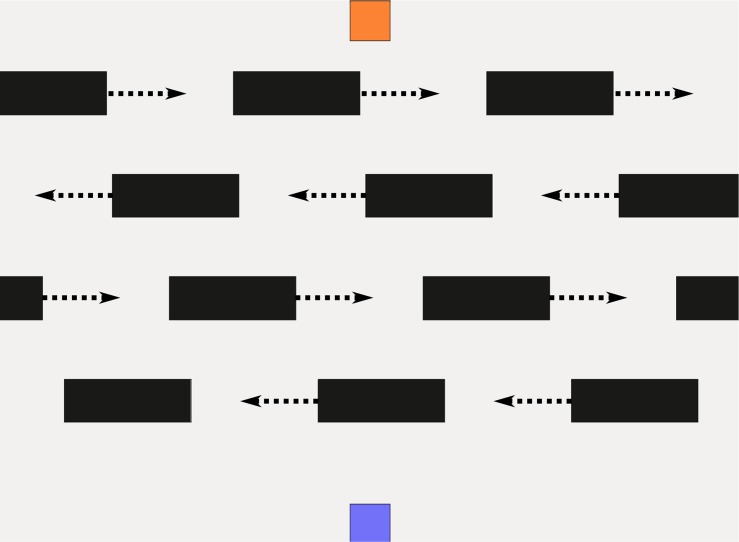
Virtual environment. Obstacles are shown in black, start position on the bottom center (blue) and the goal position at the top center (pale orange).

Within the virtual environment the smooth and straight motion is defined as the optimal solution baseline. For triggering changes in the planning horizon, a varying number of moving obstructions is integrated. With the rising number of obstacles, complexity and thus uncertainty is increased. The experiment therefore features various scenarios, which are in the following also called “levels of complexity” or “levels”. An example of the virtual environment shown to the participants is illustrated in Fig 4. Its projection is 2 m away from the input device and thus 105 cm wide and 145 cm high. All levels have the same starting field, which is always located at the bottom center and printed in a pale blue. The goal field is adversely at the top center and colored in a pale orange. The marker which is moved by the subject is represented by a dark blue box. All objects are placed on a white background, 1500 × 1100 pixel in size. At the edges, a virtual force keeps the subjects from leaving the defined workspace. Obstacles are integrated as static and moving blocks. In order to predetermine the motion of the obstacles, they only move on a straight line from left to right or vice versa. Hence, prediction is simple for a subject but becomes complex with an increasing number of these objects. Prior tests revealed that a single obstacle is very easy to circumvent. For multiple obstacles, a subject may simply wait until the straight path is free, because there is no time constraints for the task. By adding multiple obstacles that re-enter the environment, a set of moving gaps is created which makes the task considerably harder. Yet, these “obstacle lines”, as shown in Fig 4, remain easy to pass.

#### Pilot-Study for Parameter Definition

In addition to the varying number of obstacles, different velocities are used as well. The reason is that very slow obstacles are extremely easy to pass, such that a change in planning horizon might not be needed. Thus, the variance in speed and obstacle numbers allows us to investigate the planning horizon more precisely. As the number of trials rises with variability, we limit the velocities to three types, slow, medium and fast. Choosing these qualitative velocities, however, is dependent on human perception. Therefore, a pilot-study is conducted with 21 subjects (12 male and 9 female participants). All subjects are presented with an empty environment and are supposed to move three times from the start to the goal. Participants are only asked to move at slow, medium and fast velocity, which they are allowed to define themselves. Thereby, subject velocities are transformed to progression in pixel-per-frame (PPF) by factors that define the relation of measured data to size and resolution of the visualized environment.

**Fig 5:**
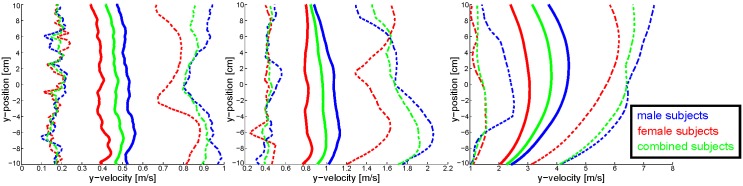
Pilot-study data of the velocities along the y-axis for the individual perception of slow, medium and fast. Red corresponds to data from female participants, blue data from male and green resembles the combined result. Solid lines represent the mean and dashed lines confine the 95% confidence interval.

Path and velocity data is collected from 21 subjects in this pilot-study prior to the experiment, in order to define acceptable obstacle velocities. For calculation of the mean velocity along the y-axis, the data is fitted and thereby smoothed with splines. Afterwards, a mean and a 95% confidence interval are calculated, see Fig 5. Women are not significantly slower than men, although the DeKiFeD does require force to be applied. Consequently, data from women and men is evaluated equally. Velocity perception is very individual, leading to a wider range of speeds. Some subjects are four times faster than others, despite equal instructions. The maximum of the combined mean is used for the fast and slow version to define boundaries. Transforming velocities to pixel-per-frame leads to 1 PPF for slow and 7 PPF for fast obstacles. Following this scheme, the medium velocity would be 2 PPF, which is just imperceptibly faster than the slow variant. Therefore, the average over all trials is chosen as a medium velocity, which leads to 3 PPF. Another important result of the pilot study is that none of the participants has difficulties in handling the input device. The DeKiFeD supplies an intuitive way of moving the virtual marker and is therefore appropriate to investigate aspects of human motion planning. With respect to the posed setup, averaging of the recorded velocities results in obstacle velocities: 1 PPF for slow, 3 PPF for medium and 7 PPF for fast. The PPF unit is chosen to resemble the progression of an obstacle over frame-rate, whereas the visualization runs with 50 frames per second (FPS).

#### Experimental Setup

**Fig 6:**
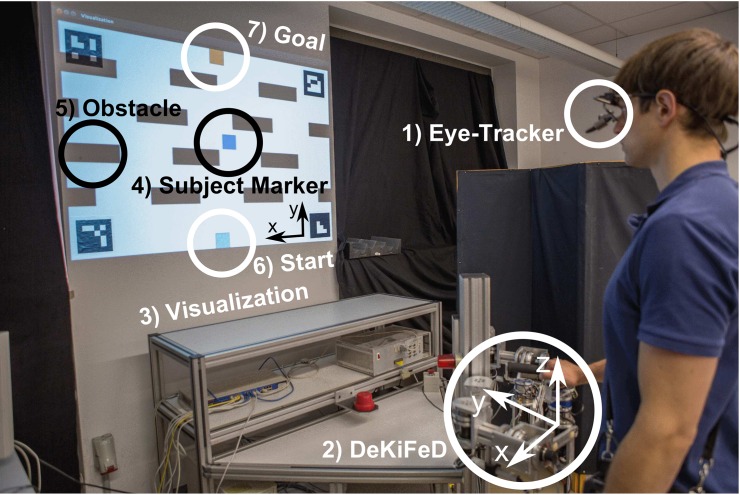
Setup of the experiment. The subject on the right wears the Dikablis Eye-Tracker (1), while using one part of the DeKiFeD (2) to move the virtual marker (4) through the virtual environment (3). The motion begins at a starting position (6) and must end at a distinct goal (7), while the subject must avoid collisions with the moving obstacles (5).

The final experimental setup is shown in Fig 6. Gaze data is recorded with a Dikablis eye-tracker [53]. After calibration the transformations between the eye-tracking device and the observed environment are retrieved. Thus, the recorded focus points are transformable to the image space with the subject’s marker position. The subject’s trajectory data may then be evaluated with respect to gaze fixation. For recording the arm motions the four DoF interface DeKiFeD is used [51]. It offers three translational and a rotational DoF within a working area of 40 *cm* × 40 *cm*. Forces applied by the user are measured with a 6-DoF force-torque sensor (JR3 Inc., Woodland, CA, USA) and transformed into acceleration and velocity of the virtual position marker. By locking the upward facing axis, subjects are restricted to move in two dimensions.

In order to gain data for different situations and with varying complexity levels, a set of ten levels is designed. Confronting subjects with a rising order of complexity probably yields significant effects, when comparing the first and the last level. However, subjects are presented with the levels in randomized order, to control for any learning effects. The ten levels have the following structure:

1. The first level is an empty field, to test what optimal trajectory subjects choose in this virtual environment.
2. The second level contains one static obstacle in the middle of the field, to cause smooth but adjusted paths.
3. The third level has one horizontal line of dynamic obstacles moving from right to left with a medium velocity.
4. In the fourth level the single line is moving diagonally from bottom right to top left, inspired by [11].
5. Two horizontal obstacle lines enter the field from the right within the fifth level, where the first and closer line moves at a slow velocity and the second one with medium velocity.
6. Equal obstacle numbers and speeds appear in the sixth level, yet the second line moves from the left to the right.
7. The seventh level is similar to the sixth, but obstacles move diagonally from bottom right to top left and the second line reversely.
8. In the eighth level, four obstacle lines are to be passed which move horizontally from the right side to the left. The first line is slow, the second and the fourth line medium and the third line fast.
9. Level nine is equal to level eight, but the second and the fourth obstacle line move from left to right.
10. Complexity is further increased in level ten as lines one and four switch from slow to medium speed, line two slows down from medium to slow speed and line three switches from fast to medium speed, if the subject gets as close as 100 pixels. In addition, line two and three also reverse their moving direction.

With this last scenario we intend to observe how subjects manage these ‘interruptions’ and the inherent uncertainty within their motion planning. The experimental task is performed twice with each level of complexity, hence resulting in a 10 (*complexity*) × 2 (*trial* − *run*) within-subjects design. Repetition of the ten levels is important to analyze the effects of learning regarding the level structure and experience with the handling of the DeKiFeD.

### Participiants

An opportunity sample of 10 female and 31 male participants, aged 18 to 33 (mean age = 24 yrs., SD = 4 yrs.) took part in the main study, five of whom were left-handed.

### Experimental Procedure

The experiments are approved by the ethics committee of the Technische Universität München and conformed to the principles expressed in the Declaration of Helsinki. A written informed consent had been obtained from all participants prior to each experiment run.

Subjects were instructed to reach the goal position whilst avoiding any obstacles but that there would be no counting of collisions and no time to beat. Afterwards, each subject was equipped with the eye-tracking system and the headset position and the alignment of eye- and field-camera were adjusted. With the subject standing at its position, the eye tracking is finally calibrated with respect to eye color and other aspects. Prior to the experiment scenarios, subjects were allowed to familiarize themselves with the handling of the DeKiFeD and the virtual environment. When the respective subject felt comfortable, the experiment started with a random level. The ten scenarios were then completed in random order twice. Upon completion of the experiment, a questionnaire was issued to every subject. The questions assess how difficult and exhausting the tasks were perceived to be by the participants.

## Main Experiment Results

Within our experiments, position and velocity data of 41 subjects is captured at 1 kHz and the corresponding eye-tracking data at 25 Hz. At first, the data is evaluated qualitatively in order to determine the effects of the level complexity. Quantitative statistical evaluation is conducted afterwards to elaborate found indications of changes in the planning horizon and to determine the probability with which the observed effects might generalize beyond the present study.

### Qualitative Data Evaluation

The following section highlights noteworthy findings and gives an interpretation with regard to the human planning horizon. The inclination and movement direction of obstacle lines seems to have negligible effects on subject behavior. For example, the scenarios four and seven, which cover diagonally moving obstacles in one and two lines, respectively, show very similar results when compared to their counterpart scenarios three and six with horizontally moving obstacles. The most relevant gaze data for the comparison of behaviors is therefore created in the levels one, two, three, six and ten. Across these levels, the complexity is raised significantly with additional obstacle lines.

**Fig 7:**
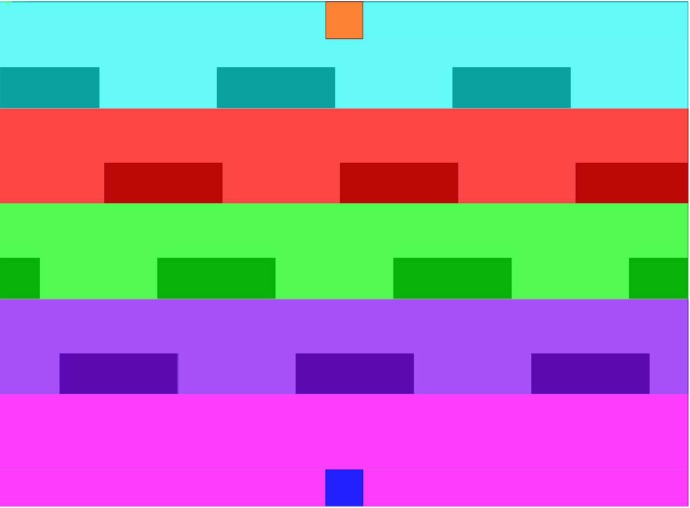
Classification zones for gaze data in a scenario with four obstacle-lines.

The subsequent qualitative evaluation considers gaze, path and velocity data of these levels, in order to find adaptations in the planning horizon of the human subjects. In order to evaluate gaze data, the virtual environment is divided into areas. One covers the start, another one the goal, and other zones are defined between the spaces start-to-first-obstacle, obstacle-to-obstacle and obstacle-to-goal. Gaze data is then distributed according to these categories such that plots reveal what the subjects focus on while moving in one of the areas. Fig 7 shows the color-based partitioning for a level with four obstacle-lines.

**Fig 8:**
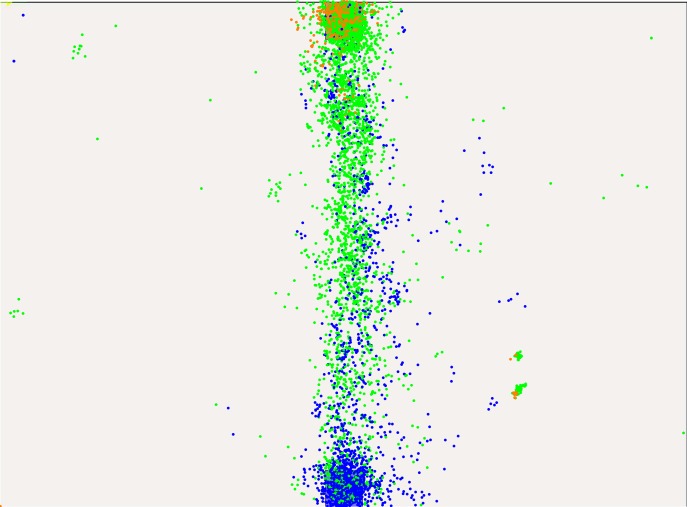
Gaze-points created in level one.

In level one, see Fig 8, subjects scan the empty area and then progress to the goal which they focus. The figure shows blue dots for gaze-points created while the subject remained at the start and green dots that represent the gaze during the motion to the goal. Clearly, subjects scan the area in front at first and the progress straight to the goal position. A planning horizon covering the full motion appears reasonable. The few orange dots indicate the gaze data recorded when the subject reaches the goal position. Across all levels we observed that subjects tend to look back. This is related to the structure of the experiment, because subjects are required to return to the starting position after each level in order to reset the system and start the next random level. Indeed, the field is free like the first level when subjects reached the goal and need to reposition to the start.

**Fig 9:**
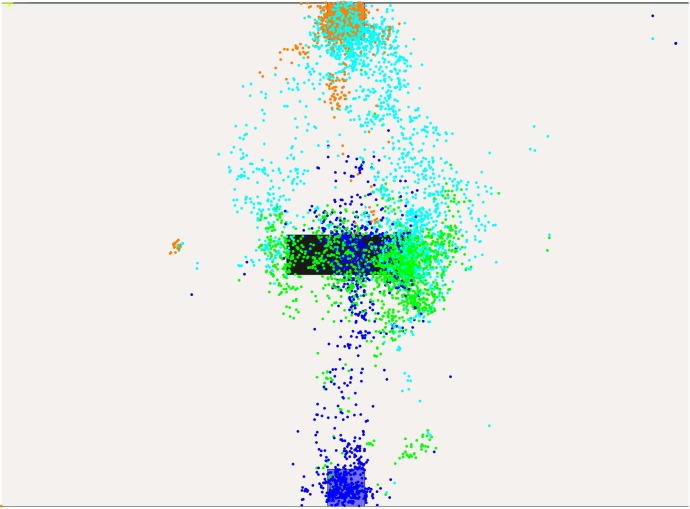
Gaze-points created in level two.

**Fig 10:**
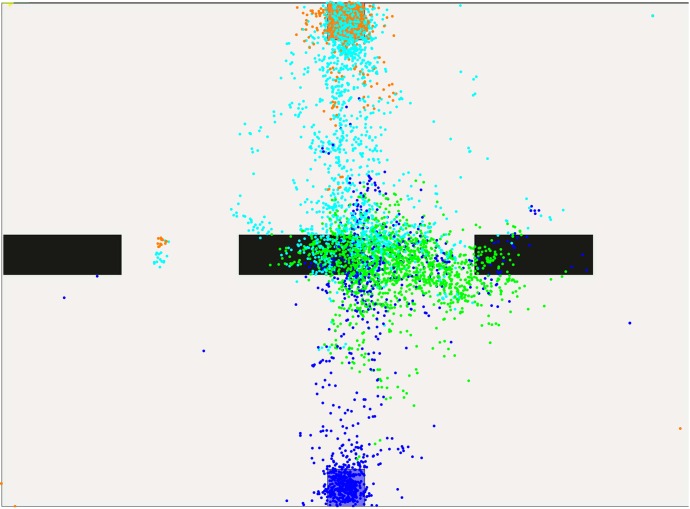
Gaze-points created in level three.

The single static obstacle in level two and the moving obstacle line in level three already receive large parts of the attention. The blue dots in Figs 9 and 10 indicate that the scanning of the area ends mostly at the obstacle. Yet, it is possible that the remaining area is processed by the peripheral field of view. The green dots now correspond to the area between the start position and the first obstacle. Most of the gaze is dedicated to the obstacles, although it is not moving in level two. Once the obstacle is reached, gaze slowly shifts to the goal, as the teal dots indicate. Similarly to the problem posed in the Problem Description section, humans seem to reduce their planning horizon to the most immediate obstacle at first. However, position and velocity data will show that subjects perform very smooth trajectories in level two and are able to stick closely to the optimal straight path in level three.

**Fig 11:**
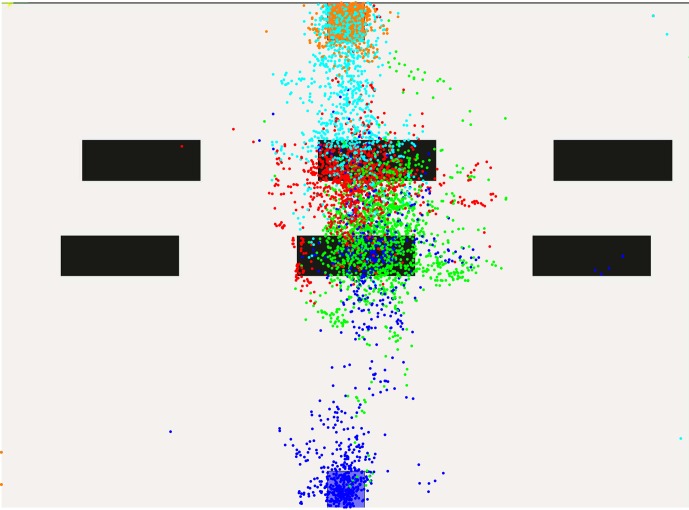
Gaze-points created in level five.

**Fig 12:**
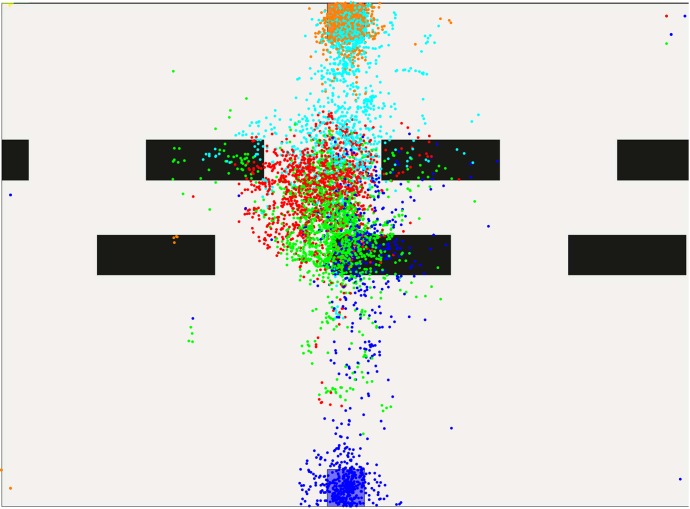
Gaze-points created in level six.

In the following levels, gaze data shows even less look-ahead. Even in the scenarios with only two lines, attention is mostly on the obstacles. Figs 11 and 12 illustrate this. Red dots now cover the area between the two obstacle lines, all other colors are assigned as before. Notably, the red and green gaze points are mostly between the obstacles which are the most immediate at that position. Since the goal or the area past the obstacles is not in the focus, we assume that the planning horizon is constrained to find a solution for passing the obstacles.

**Fig 13:**
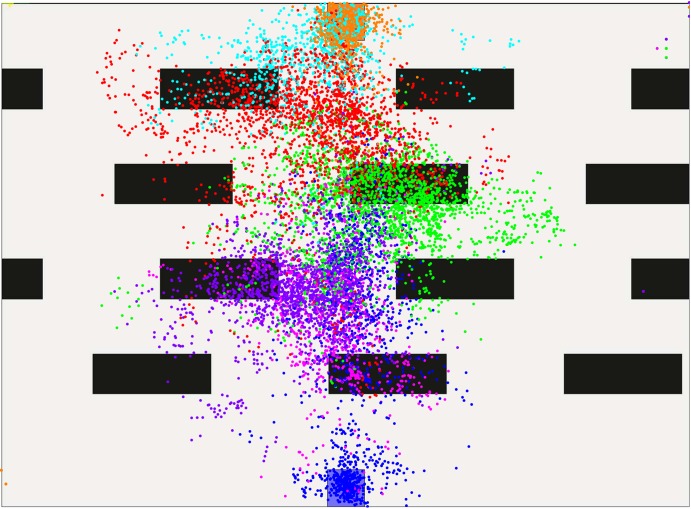
Gaze-points created in level nine.

**Fig 14:**
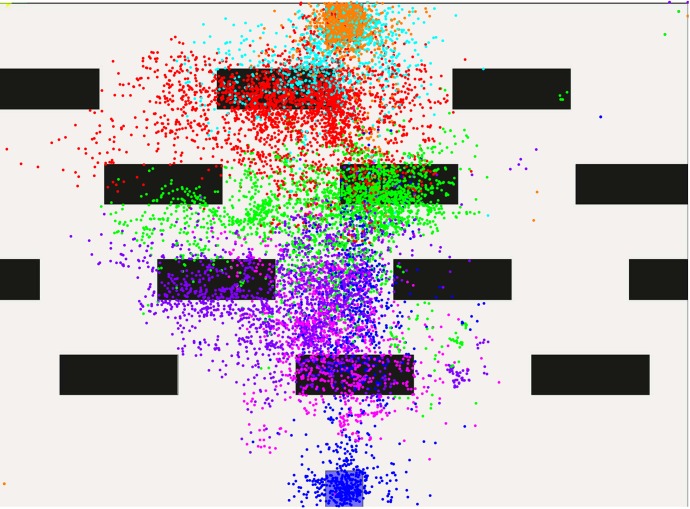
Gaze-points created in level ten.

These effects become even stronger with four obstacle lines. The color-to-area alignment is now blue-pink-violet-green-red-teal-orange covering the spaces from start to goal. In Figs 13 and 14, gaze-points illustrated in pink cover the first two obstacle lines, proposing a larger planning horizon. Violet, red and green dots, however, are again constrained to the areas between obstacles, indicating no further looking-ahead. A comparison of level one and nine or ten suggests, that humans do reduce their planning horizon. Yet, this behavior may be also interpreted as a motion towards the most immediate obstacle which is then passed. In fact, further areas might be covered by the peripheral vision of the subject.

**Fig 15:**
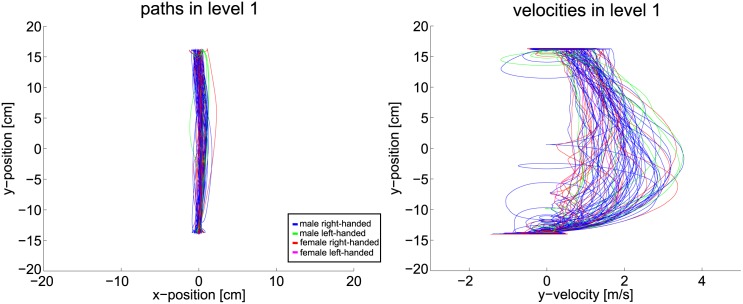
Path and velocity data of level one.

Therefore, path and velocity data must be considered to gain insight how far the planning horizon reaches. Following plots are color coded such that blue depicts male right-handed subjects, green depicts male left-handed subjects, red depicts female right-handed subjects and pink is used for female left-handed subjects. Handedness, however, does not have any formative influence on the results. Fig 15 shows the position and velocity data of the first scenario. Without any obstructions the path and velocity are smooth and lead directly from start to goal. This supports the proposition that the planning horizon follows the OC idea for this simple level.

**Fig 16:**
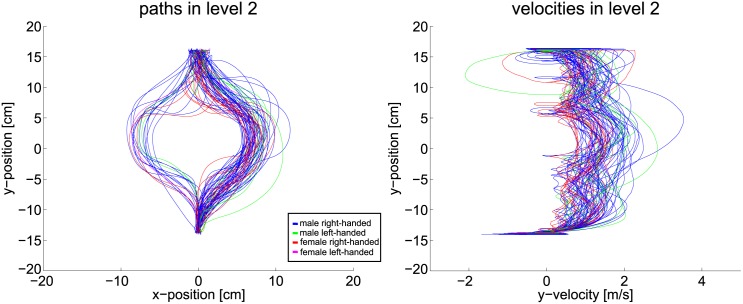
Path and velocity data of level two.

**Fig 17:**
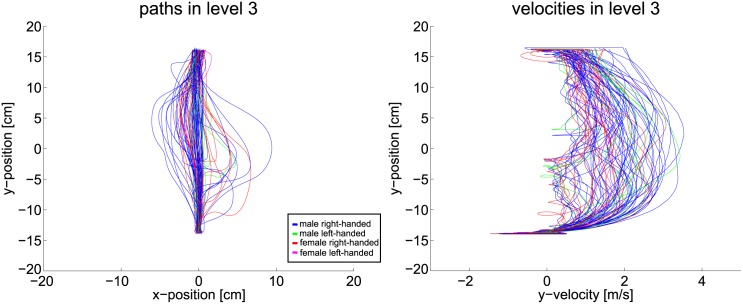
Path and velocity data of level three.

The single static obstacle in scenario two is easily circumvented by all subjects with smooth paths, see Fig 16. This argues against the shorter planning horizon. Velocity data shows that subjects brake in front of the obstacle and speed up to go around it. A smooth and continuous progression of the velocity is expected but it appears that the planning horizon does not cover the full distance to the goal. Visible loops in the velocities originate from collisions, where the marker is stopped and moves backwards slightly until new speed is gained. In scenario three, see Fig 17, the single obstacle line does also not pose a problem for most subjects. Both path and velocity remain smooth with some visible braking in the velocity plot. This braking combined with the fact that most subjects moved straight towards the obstacle at first, allows for the assumption that some participants relied on a shorter planning horizon. Yet, the majority of the subjects follows the shortest path solution and achieves smooth progression.

**Fig 18:**
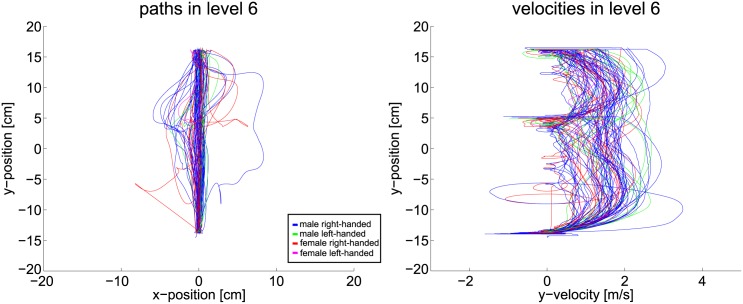
Path and velocity data of level six.

For the case of two obstacle lines, velocity and path remain mostly smooth. Fig 18 visualizes the data captured in level six. Indeed, as the gaze-points revealed, many subjects are not able to surpass both obstacle lines at once. Their planning horizon seems restricted to the area between the obstacles as the braking in the center of the velocity plot reveals. Yet, the majority of the subjects follows the shortest path and produces smooth velocities.

**Fig 19:**
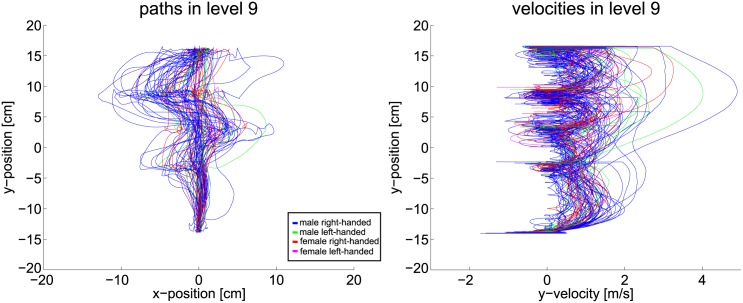
Path and velocity data of level nine.

**Fig 20:**
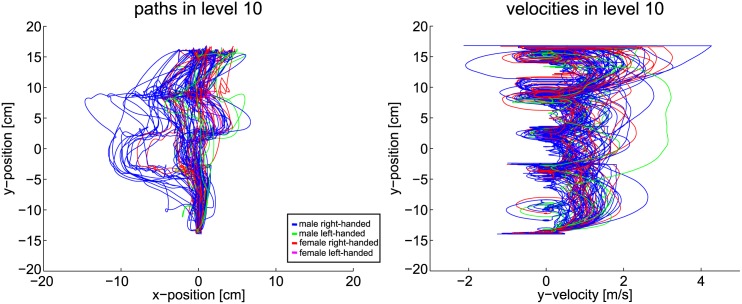
Path and velocity data of level ten.

The scenarios nine and ten add another two obstacle rows to the environment. With the added complexity a change in the behavior must be visible, if the hypothesis about the correlation of uncertainty and planning horizon holds. In fact, the data changes drastically as Figs 19 and 20 show. With the complexity of the ninth level, subjects are often not able to apply smooth paths. The goal-directed motion is therefore reduced to stepwise progression. Path data deviates strongly from the shortest path solution. Velocity data reveals an increase in braking, especially in front of the third and fourth obstacle line. Thus, smoothness of the trajectories is diminished. Taking into account gaze data, the hypothesis holds that subjects are not able to plan a path directly to the goal. Clearly, subjects progress by passing one obstacle line after the other. Indeed, two subjects noticed and used the free optimal path which appears after 2s. If the hypothesis holds that humans reduce their planning horizon within uncertain situations, the observed effects of level nine must intensify if further complexity is added. Thus, in level ten the obstacles additionally change their velocity and direction of movement. The extra uncertainty actually enhances the mentioned aspects even more, as Fig 20 shows. Paths now differ even more from the straight path. The velocity data contains increased signs of braking and collisions.

This qualitative data evaluation substantiates the assumption that humans alter their planning horizon in order to cope with complex environments. Subjects focus the most immediate obstacle and pass it before the next obstruction is considered. Further, subjects deviate from the shortest path and omit the global optimum. The velocity data also suggests that the subjects are not able to plan far ahead in complex environments, in order to avoid braking between obstacle lines. In the following, these results are further elaborated by statistical evaluations

### Statistical Data Evaluation

The statistical evaluation of the acquired data focuses on two distinct parameters. Firstly, the visual look-ahead is considered, where the position of the marker in the virtual environment is compared with the focus of synchronized gaze. Secondly, the velocity profile is analyzed because smooth velocities indicate a continuous motion and thus a large planning horizon and vice versa. Finally, the deviation from the optimal path is considered, whereby smaller deviations would indicate a larger planning horizon.

#### Visual Look-Ahead

One indicator of the planning horizon in the experiment may be the distance between the human’s visual focus of attention and the position of the participant’s cursor. Hence, in order to ascertain whether the planning horizon changed with increasing complexity of the scene, eye tracking data were evaluated in combination with position data on the participants’ cursor. Specifically, the mean distance between participants’ visual focus and their cursor’s position was compared over the different complexity levels. Due to missing data, 10 data-sets were excluded from the analysis, leaving a sample of N = 31. Of the 620 remaining values, 25 individual missing values (= 4.05% of the data-set) were replaced by the group mean.

A repeated-measures 10 (*complexity*) × 2 (*trial* − *run*) ANOVA showed a non-significant (accepted *α*-level *p* = .05) run main effect (*F*(1, 30) = 0.13, *p* = .73), indicating that over all levels of complexity, the distance between the participants’ cursor and the point of visual fixation did not vary significantly between the first and second run. The ANOVA further showed a small but significant main effect of complexity on the mean fixation-cursor distance (*F*(5.73, 171.86*) = 4.82, *p* < .001, *η*_p_^2^ = .14, *with Greenhouse-Geisser correction). The mean values indicate a trend of increasingly smaller distances with increasing level complexity. Post-hoc contrasts to the baseline (level 1) confirm that the distance is significantly smaller in most complexity levels (with the exception of levels 2 and 5) compared to the visual behavior shown in the fully observable environment in level 1. Mean values, standard errors and the results of the post-hoc contrasts to the baseline (level 1) are summarized in Table 1.

**Table 1:** Mean values and standard errors for average mean distances between eye fixation and cursor position (in pixels).

	Levels of Complexity	Mean (SE)	Baseline Contrast
1	No objects	300.279 (17.733)	–
2	1 static object	264.118 (18.731)	*F*(1, 30) = 2.02 *p* = .166
3	1 obstacle line moving horizontally	244.169 (12.854)	*F*(1, 30) = 9.01 *p* = .005*, *η*_p_^2^ = .23
4	1 obstacle line moving diagonally	250.484 (11.972)	*F*(1, 30) = 7.63 *p* = .010*, *η*_p_^2^ = .20
5	2 obstacle lines moving horizontally at different speeds in same direction	285.741 (24.006)	*F*(1, 30) = 0.45 *p* = .510
6	2 obstacle lines moving horizontally at different speeds in opposite directions	240.153 (12.529)	*F*(1, 30) = 10.43 *p* = .003**, *η*_p_^2^ = .26
7	2 obstacle lines moving diagonally at different speeds in opposite directions	262.189 (16.754)	*F*(1, 30) = 4.42 *p* = .044*, *η*_p_^2^ = .13
8	4 obstacle lines moving horizontally at different speeds in same direction	206.796 (16.055)	*F*(1, 30) = 18.16 *p* < .001**, *η*_p_^2^ = .34
9	4 obstacle lines moving horizontally at different speeds in opposite directions	228.984 (25.359)	*F*(1, 30) = 6.00 *p* = .020*, *η*_p_^2^ = .17
10	4 obstacle lines moving horizontally at different speeds in opposite directions with both changing	189.679 (11.858)	*F*(1, 30) = 25.08 *p* < .001**, *η*_p_^2^ = .46

*sig. at *p* < .05

**sig. at Bonferroni corrected *p* < .005

Bonferroni-adjusted post-hoc paired-samples t-tests that compared the visual look-ahead between levels 3, 6 and 10 indicate that in comparison to the most complex level 10, subjects looked ahead of their cursor a significantly longer distance in the less complex levels 3 (*t*(30) = 2.86, *p* < .016) and 6 (*t*(30) = 2.60, *p* < .016), whereas the difference between levels 3 and 6 was not significant at the adjusted *α*-level of *p* = .016(*t*(30) = 0.22, *p* = .83). Thus, the statistical analyses indicate that the planning horizon, as indicated by the visual look-ahead, shortens with increasing scene complexity.

#### Smoothness of Velocity

Further analyses investigated the effects of scenario complexity on participants’ variation of movement velocity. Using Roy’s largest root, a repeated-measures MANOVA indicated a significant medium-sized main effect of complexity on velocity variation on x- and y-axis (Θ = 2.62, *F*(9, 351) = 102.02, *p* < .001, *η*_p_^2^ = .72). Follow-up ANOVA showed that on both axes, velocity varied significantly between the different complexity levels with large and medium effect sizes on the x- and y- axis, respectively (x-axis: *F*(9, 351) = 99.17, *p* < .001, *η*_p_^2^ = .72; y-axis: *F*(9, 351) = 17.95, *p* < .001, *η*_p_^2^ = .32). There was also a large and significant multivariate main effect of run (Θ = 3.74, *F*(2, 38) = 70.97, *p* < .001, *η*_p_^2^ = .79), and a small yet significant interaction effect (Θ = 0.18, *F*(9, 351) = 7.04, *p* < .001, *η*_p_^2^ = .15). Looking at the mean values, it seems that the velocity variation on the x-axis was significantly larger on the first run than the second run, while it was approximately similar between runs on the y-axis.

Mean values indicate a tendency towards greater velocity variations on the x-axis, but not the y-axis in levels with higher complexity compared to levels with lower complexity. Noticeable difference is level 2, which contains 1 static object and seems to encourage more extreme x-axis velocity maneuvers. Post-hoc contrasts to the baseline indicate that the velocity variation on the x-axis is significantly smaller in the baseline condition (level 1) compared to movements in all other levels. On the other hand, velocity variation on the y-axis is significantly larger in the baseline condition compared to the variation observed in levels 2 and 5-10. Tables 2 and 3 summarize the results of the baseline contrasts.

**Table 2:** Mean values and standard errors for average velocity variation on x-axis (in dm/s).

	Levels of Complexity	Mean (SE)	Baseline Contrast
1	No objects	.068 (.005)	–
2	1 static object	.481 (.021)	*F*(1, 39) = 483.75 *p* < .001**, *η*_p_^2^ = .93
3	1 obstacle line moving horizontally	.152 (.012)	*F*(1, 39) = 37.50 *p* < .001**, *η*_p_^2^ = .49
4	1 obstacle line moving diagonally	.168 (.011)	*F*(1, 39) = 58.07 *p* < .001**, *η*_p_^2^ = .60
5	2 obstacle lines moving horizontally at different speeds in same direction	.123 (.006)	*F*(1, 39) = 56.95 *p* < .001**, *η*_p_^2^ = .59
6	2 obstacle lines moving horizontally at different speeds in opposite directions	.127 (.012)	*F*(1, 39) = 20.11 *p* < .001**, *η*_p_^2^ = .34
7	2 obstacle lines moving diagonally at different speeds in opposite directions	.174 (.012)	*F*(1, 39) = 60.51 *p* < .001**, *η*_p_^2^ = .61
8	4 obstacle lines moving horizontally at different speeds in the same direction	.283 (.013)	*F*(1, 39) = 216.36 *p* < .001**, *η*_p_^2^ = .85
9	4 obstacle lines moving horizontally at different speeds in opposite directions	.292 (.018)	*F*(1, 39) = 148.69 *p* < .001**, *η*_p_^2^ = .79
10	4 obstacle lines moving horizontally at different speeds in opposite directions with both changing	.260 (.013)	*F*(1, 39) = 229.54 *p* < .001**, *η*_p_^2^ = .85

**sig. at Bonferroni corrected *p* < .005

**Table 3:** Mean values and standard errors for average velocity variation on y-axis (in dm/s).

	Levels of Complexity	Mean (SE)	Baseline Contrast
1	No objects	.685 (.034)	–
2	1 static object	.514 (.026)	*F*(1, 39) = 42.39 *p* < .001**, *η*_p_^2^ = .52
3	1 obstacle line moving horizontally	.671 (.039)	*F*(1, 39) = 0.23 *p* = .634
4	1 obstacle line moving diagonally	.702 (.046)	*F*(1, 39) = 0.27 *p* < .609
5	2 obstacle lines moving horizontally at different speeds in same direction	.623 (.036)	*F*(1, 39) = 5.05 *p* < .030*, *η*_p_^2^ = .12
6	2 obstacle lines moving horizontally at different speeds in opposite directions	.578 (.032)	*F*(1, 39) = 18.38 *p* < .001**, *η*_p_^2^ = .32
7	2 obstacle lines moving diagonally at different speeds in opposite directions	.543 (.033)	*F*(1, 39) = 25.2 *p* < .001**, *η*_p_^2^ = .39
8	4 obstacle lines moving horizontally at different speeds in the same direction	.559 (.031)	*F*(1, 39) = 19.87 *p* < .001**, *η*_p_^2^ = .34
9	4 obstacle lines moving horizontally at different speeds in opposite directions	.504 (.030)	*F*(1, 39) = 36.58 *p* < .001**, *η*_p_^2^ = .48
10	4 obstacle lines moving horizontally at different speeds in opposite directions with both changing	.512 (.022)	*F*(1, 39) = 41.60 *p* < .001**, *η*_p_^2^ = .52

*sig. at *p* < .05

**sig. at Bonferroni corrected *p* < .005

Bonferroni-adjusted post-hoc paired-samples t-tests comparing the x- and y-axis velocity variation between levels 3, 6 and 10 indicate that in comparison to the most complex level 10, subjects varied their movement speed on the x-axis significantly less in level 3 (*t*(40) = −7.72, *p* < .001) and level 6 (*t*(40) = −6.62, *p* < .001), whereas the difference between levels 3 and 6 was not significant at the adjusted *α*-level of p = .016 (*t*(40) = 2.15, *p* = .04). In contrast, y-axis velocity variation increased significantly with increasing velocity (levels 3 vs. 6: *t*(40) = 3.33, *p* < .016; levels 6 vs. 10: *t*(39) = 2.65, *p* < .016; levels 3 vs. 10: *t*(39) = 5.83, *p* < .001).

#### Deviation from an Optimal Path

In order to investigate the extent to which the level of complexity affects a deviation from the optimal path, the absolute mean values of participants’ average path deviations were examined. Mean values and standard deviations are shown in Table 4. The mean values indicate, that on average, participants deviated from the optimum path the most in level 2 with one immobile object, followed by large deviations in levels with four objects. The smallest deviations from the optimum path are observed in the baseline level 1, followed by those levels that contain 1 and 2 objects, with the exception of level 2. The large deviations in level 2 may be attributed to the fact that there are two optimum paths around the object. Overall, a large variance can be observed in the average deviations, whereby the variance seems to increase with the number of objects in the scene.

**Table 4:** Mean values and standard errors for the mean deviations from the optimal path (in dm).

	Levels of Complexity	Mean (SE)	Baseline Contrast
1	No objects	.020 (.002)	–
2	1 static object	.244 (.006)	*F*(1, 40) = 1374.62 *p* < .001**, *η*_p_^2^ = .97
3	1 obstacle line moving horizontally	.061 (.008)	*F*(1, 40) = 23.19 *p* < .001**, *η*_p_^2^ = .37
4	1 obstacle line moving diagonally	.078 (.008)	*F*(1, 40) = 43.98 *p* < .001**, *η*_p_^2^ = .52
5	2 obstacle lines moving horizontally at different speeds in same direction	.052 (.007)	*F*(1, 40) = 19.36 *p* < .001**, *η*_p_^2^ = .33
6	2 obstacle lines moving horizontally at different speeds in opposite directions	.053 (.008)	*F*(1, 40) = 13.56 *p* < .001**, *η*_p_^2^ = .25
7	2 obstacle lines moving diagonally at different speeds in opposite directions	.060 (.008)	*F* (1, 40) = 19.82 *p* < .001**, *η*_p_^2^ = .33
8	4 obstacle lines moving horizontally at different speeds in the same direction	.170 (.022)	*F*(1, 40) = 46.71 *p* < .001**, *η*_p_^2^ = .54
9	4 obstacle lines moving horizontally at different speeds in opposite directions	.107 (.011)	*F*(1, 40) = 56.68 *p* < .001**, *η*_p_^2^ = .59
10	4 obstacle lines moving horizontally at different speeds in opposite directions with both changing	.140 (.021)	*F*(1, 40) = 31.82 *p* < .001**, *η*_p_^2^ = .44

**sig. at Bonferroni corrected *p* < .005

Repeated measures 10 (*complexity*) × 2 (*trial* − *run*) ANOVA revealed a significant main effect of run (*F*(1, 40) = 20.55, *p* < .001) and complexity (*F*(3.73, 149.08) = 42.88, *p* < .001), but no significant interaction (*F*(4.02, 160.93) = 0.84, *p* = .50). Looking at the mean values, the data thus indicate that participants deviated significantly less from the optimum path when moving through the levels for a second time compared to the first time. Presumably, this can be attributed to a reduced uncertainty. Regarding the complexity, post-hoc comparisons to the baseline level 1 confirmed that participants deviated significantly less in the baseline condition compared to each of the 9 experimental levels, see Table 4. The contrasts further indicate that the deviations were considerably larger in levels with four objects compared to those with only two objects.

Bonferroni-adjusted post-hoc paired-samples t-tests which compared the mean deviations from the optimal path between levels 3, 6 and 10 indicate that in comparison to the most complex level 10, subjects deviated from the path significantly less in the less complex levels 3 (*t*(40) = −4.09, *p* < .001) and 6 (*t*(40) = −4.98, *p* < .001), whereas the difference between levels 3 and 6 was not significant at the adjusted *α*-level of *p* = .016(*t*(40) = 1.18, *p* = .25). Thus, the statistical analyses indicate that the deviation from the optimal path increases with increasing scene complexity; however, this effect is only significant with high levels of complexity.

Looking at the maximum deviations from the optimum path, a similar pattern is observed, with the largest deviations occurring in level 2, followed by the levels with four objects. The smallest maximum deviations are found in the baseline level, suggesting that participants followed the optimum trajectory when there are no objects to circumvent. On the other hand, with the introduction of further objects, participants increasingly deviated from the optimum path. Again, the variance seems to increase notably with the introduction of four moving objects, suggesting that individual differences effect more variance in the movement with increasing scene complexity, while individual difference are much less notable in the levels with lower scene complexity, in particular the baseline level 1.

Repeated measures 10 (*complexity*) × 2 (*trial* − *run*) ANOVA revealed a significant main effect of run (*F*(1, 40) = 26.80, *p* < .001) and complexity (*F*(3.39, 135.49) = 68.12, *p* < .001) on the maximum deviations, but no significant interaction (*F*(4.94, 197.64) = 0.65, *p* = .66). Looking at the mean values, the data thus indicate that participants deviated significantly less from the optimum path when moving through the levels for a second time compared to the first time. Presumably, this can be attributed to a reduced uncertainty. Regarding the complexity, post-hoc comparisons to the baseline level 1 confirmed that participants deviated significantly less in the baseline condition compared to each of the 9 experimental levels, see Table 5. Contrasts further confirmed that the deviations were significantly larger in levels with four objects compared to those with only two objects.

**Table 5:** Mean values and standard errors for the maximum deviations from the optimal path (in dm).

	Levels of Complexity	Mean (SE)	Baseline Contrast
1	No objects	.069 (.005)	–
2	1 static object	.764 (.015)	*F*(1, 40) = 2422.73 *p* < .001**, *η*_p_^2^ = .98
3	1 obstacle line moving horizontally	.205 (.023)	*F*(1, 40) = 31.27 *p* < .001**, *η*_p_^2^ = .44
4	1 obstacle line moving diagonally	.241 (.025)	*F*(1, 40) = 42.08 *p* < .001**, *η*_p_^2^ = .51
5	2 obstacle lines moving horizontally at different speeds in same direction	.162 (.016)	*F*(1, 40) = 25.98 *p* < .001**, *η*_p_^2^ = .39
6	2 obstacle lines moving horizontally at different speeds in opposite directions	.178 (.023)	*F*(1, 40) = 19.10 *p* < .001**, *η*_p_^2^ = .32
7	2 obstacle lines moving diagonally at different speeds in opposite directions	.240 (.026)	*F*(1, 40) = 38.35 *p* < .001**, *η*_p_^2^ = .49
8	4 obstacle lines moving horizontally at different speeds in the same direction	.598 (.062)	*F*(1, 40) = 67.84 *p* < .001**, *η*_p_^2^ = .63
9	4 obstacle lines moving horizontally at different speeds in opposite directions	.484 (.038)	*F*(1, 40) = 110.17 *p* < .001**, *η*_p_^2^ = .73
10	4 obstacle lines moving horizontally at different speeds in opposite directions with both changing	.456 (.040)	*F*(1, 40) = 87.19 *p* < .001**, *η*_p_^2^ = .69

**sig. at Bonferroni corrected *p* < .005

As it was the case with the other measures that were used to infer changes in the planning horizon, Bonferroni-adjusted post-hoc paired-samples t-tests which compared the maximum deviations from the optimal path between levels 3, 6 and 10 indicate that in comparison to the most complex level 10, subjects deviated from the path significantly less in the less complex levels 3 (*t*(40) = −6.50, *p* < .001) and 6 (*t*(40) = −6.87, *p* < .001), whereas the difference between levels 3 and 6 was not significant at the adjusted *α*-level of p = .016 (*t*(40) = 1.42, *p* = .16). Thus, the statistical analyses corroborates the finding on the mean path deviation which is that the deviation from the optimal path increases with increasing scene complexity, whereby this effect only seems to take effect with high levels of complexity.

## Discussion and Conclusions

In summary, a literature review indicated that many recent optimal control and model predictive control methods can not provide an accurate account of human locomotion, specifically for avoidance behaviors that originate from suddenly emerging collision situations. Instead, it was stipulated that a nonlinear model predictive control-based framework might depict human locomotion behavior in some circumstances more accurately. Hence, the goal of the present work was to investigate human locomotion planning processes from a control method-based point of view, to provide an empirical basis for the development of predictive models that might improve human interactions with mobile robots. Specifically, it was explored whether the planning horizon for locomotion, i.e. the physical space that humans subconsciously take into consideration when planning their movement paths, changes with the complexity of the presented environment. Environment complexity was varied by systematically changing the number of present obstacles as well as the number of differing object velocities and the objects’ movement directions, that subjects were asked to avoid in each experimental run whilst moving their cursor between two locations. Since the cognitive processes that are involved in determining the planning horizon cannot be directly observed, three behavioral measures were investigated which are assumed to be indicators of the planning horizon. These are visual look-ahead, smoothness of the performed trajectory and deviations from the optimal path. Qualitative and quantitative analyses of the experimental results indicate that humans adapt their planning horizon, as indicated by the behavioral measures, with increasing environment complexity. If the environment is free of obstacles, the subjects’ gaze tends to scan the whole area between start and goal position. Paths and velocities of the motion are then smooth and follow a straight line. With a static obstacle, subjects focus on the obstacle to avoid collisions but still produce smooth trajectories. If the complexity is further increased by adding more obstacles and varying speed and direction of the obstacles, gaze tends to address only the next obstacle to be passed.

To conclude this work, the results from simulations and experiments are discussed. With respect to literature, the simulations of the presented nonlinear model predictive control approach for human locomotion prediction revealed very similar effects regarding avoidance behaviors. While optimal control models the human desire for minimum effort in path planning and trajectory execution, the reduced planning horizon within nonlinear model predictive control resembles more accurately the avoidance movements presented within literature. The simulation results illustrate the influence of a changing planning horizon for locomotion prediction. Accordingly, the change in planning horizon is understood as a human behavior and thus investigated in the designed experiment. Results obtained in the experiment validate the control approach and support the integration of this aspect within prediction methods. A detailed model for human motion planning behavior with an adaptive planning horizon is not obtained from the experiment. Indeed, the experimental setup and its result pose a strong foundation and motivation to further investigate the human planning horizon. Changing the planning horizon poses one factor that is not yet considered in current models. However, there are various aspects which may affect the trajectories similarly and require further studies based on present results.

Regarding the conducted experiment, the recorded data show smooth paths and velocities up to two lines of moving obstacles. This performance declines if more obstacles are to be passed. Most subjects then progress in small physical increments, which leads to noticeable braking, as indicated by the velocity data. The statistical evaluations support these findings. Evaluation of the mean distance between the marker and the gaze position shows that looking ahead diminishes when comparing an empty scenario with a very complex scenario. Furthermore, when considering the shortest path as the optimal solution, subjects deviate significantly from this solution if the environment is complex.

While the presented results provide a clear indication that human locomotion planning changes with increasing environment complexity and illuminates to some degree the nature of these changes, further studies are required to determine the extent to which these findings might generalize to three-dimensional environments with varying stimuli and different locomotion behaviors. For instance, in the present experiments, the environment was seen from a bird’s-eye point of view, where all obstacles that might affect the planned path are visible and their movements predictable. Hence, the subjects had much more knowledge of possible path obstructions than would be the case in a crowded real-world environment, where moving obstacles are oftentimes partially occluded and their movements more difficult to predict. Moreover, assuming that avoidance-motivated locomotion planning is in humans strongly influenced by visual input, it would seem likely that obstacles of differing visual salience might affect human locomotion planning (e.g. the planning horizon might be more likely to extend to a large, red object than a small, grey object in a visually cluttered environment). Furthermore, the cognitive mechanisms that affect the planning horizon are still unclear. While subjects rated the experimental task overall as undemanding and effortless, it seems likely that cognitive load increased with increasing environment complexity. However, the experiment was neither designed nor destined to uncover which features contributed most to the increase in cognitive load: the number of obstacles, the speed with which they traveled or the path they took. A detailed and systematic analysis of differential effects of obstacle features on the planning horizon thus remains subject to future investigations. Similarly, it must still be investigated whether analog effects on the planning horizon can be observed with regard to other human motions, in particular walking. Further experiments are therefore needed to evaluate and adjust existing prediction models or serve as an empirical basis to develop new models. However, with respect to shown simulations and results, the detailed knowledge about human locomotion behavior seems to hold the potential to improve current and future model-based locomotion prediction algorithms. Future applications of robots performing collaborative tasks in shared environments will benefit from improved predictions. Robots moving in warehouses or production halls will entail less disturbance for nearby humans and pose efficient as well as convenient collaboration partners.

## References

[1] Lasota P, Shah J. Analyzing the effects of human-aware motion planning on close-proximity human–robot collaboration. Human Factors: The J. of the Human Factors and Ergonomics Society. 2015;57(1):21–33. 10.1177/0018720814565188 25790568 PMC4359211

[2] Mainprice J, Hayne R, Berenson D. Predicting human reaching motion in collaborative tasks using inverse optimal control and iterative re-planning. In: Int. Conf. on Robotics and Automation; 2015. p. 885–892.

[3] Lefèvre S, Vasquez D, Laugier C. A survey on motion prediction and risk assessment for intelligent vehicles. Robomech J. 2014;1(1):1–14. 10.1186/s40648-014-0001-z

[4] Albrecht S, Basili P, Glasauer S, Leibold (Sobotka) M, Ulbrich M. Modeling and Analysis of Human Navigation with Crossing Interferer Using Inverse Optimal Control. In: Int. Conf. on Mathematical Modelling; 2012. p. 158–163.

[5] Elnagar A. Prediction of moving objects in dynamic environments using Kalman filters. In: Int. Symp. on Computational Intelligence in Robotics and Automation; 2001. p. 414–419.

[6] Ziebart BD, Ratliff N, Gallagher G, Mertz C, Peterson K, Bagnell JA, et al. Planning-based Prediction for Pedestrians. In: Int. Conf. on Intelligent Robotics and Systems; 2009. p. 3931–3936.

[7] Arechavaleta G, Laumond JP, Hicheur H, Berthoz A. An optimality principle governing human walking. Trans. on Robotics. 2008;24(1):5–14. 10.1109/TRO.2008.915449

[8] Mombaur K, Truong A, Laumond JP. From Human to Humanoid Locomotion—An inverse optimal control approach. Autonomous Robots. 2009;28(3):369–383. 10.1007/s10514-009-9170-7

[9] Papadopoulos AV, Bascetta L, Ferretti G. Generation of Human Walking Paths. In: Int. Conf. on Intelligent Robots and Systems; 2013. p. 1676–1681.

[10] Basili P, Sağlam M, Kruse T, Huber M, Kirsch A, Glasauer S. Strategies of locomotor collision avoidance. Gait & Posture. 2013;37(3):385–390. 10.1016/j.gaitpost.2012.08.003 22975461

[11] Huber M, Su YH, Krüger M, Faschian K, Glasauer S, Hermsdörfer J. Adjustments of speed and path when avoiding collisions with another pedestrian. PloS One. 2014;9(2). 10.1371/journal.pone.0089589 PMC3935867 24586895

[12] Tastan B, Sukthankar G. Leveraging human behavior models to predict paths in indoor environments. Pervasive and Mobile Computing. 2011;7(3):319–330. 10.1016/j.pmcj.2011.02.003

[13] Kaelbling L, Littman M, Moore A. Reinforcement Learning: A Survey. J. of Artificial Intelligence Research. 1996;p. 237–285.

[14] Dragan A, Srinivasa S. Generating Legible Motion. In: Robotics: Science and Systems; 2013.

[15] Reich BD. An architecture for behavioral locomotion. IRCS Technical Reports Series. 1997;p. 51.

[16] Ahmadi-Pajouh MA, Towhidkhah F, Gharibzadeh S, Mashhadimalek M. Path planning in the hippocampo-prefrontal cortex pathway: An adaptive model based receding horizon planner. Medical Hypotheses. 2007;68(6):1411–1415. 10.1016/j.mehy.2006.06.060 17337125

[17] Carton D, Turnwald A, Wollherr D, Buss M. Proactively approaching pedestrians with an autonomous mobile robot in urban environments In: Experimental Robotics. Springer; 2012 p. 199–214. 10.1007/978-3-319-00065-7_15

[18] Olivier AH, Marin A, Crétual A, Pettré J. Minimal predicted distance: A common metric for collision avoidance during pairwise interactions between walkers. Gait & Posture. 2012;36(3):399–404. 10.1016/j.gaitpost.2012.03.021 22560717

[19] Ondřej J, Pettré J, Olivier AH, Donikian S. A synthetic-vision based steering approach for crowd simulation. Trans. on Graphics. 2010;29(4):123.

[20] Fajen BR, Warren WH. Behavioral dynamics of steering, obstacle avoidance, and route selection. Experimental Psychology: Human Perception and Performance. 2003;29(2):343. 10.1037/0096-1523.29.2.343 12760620

[21] Goffman E. Relations in public: Microstudies of the public order. Harper and Row, New York; 1971.

[22] Nilsson NJ. Shakey the robot. SRI Int Technical Note. 1984;325.

[23] Bitgood S, Dukes S. Not another step! Economy of movement and pedestrian choice point behavior in shopping malls. Environment and behavior. 2006;38(3):394–405. 10.1177/0013916505280081

[24] Sparrow WA, Newell KM. Metabolic energy expenditure and the regulation of movement economy. Psychonomic Bulletin & Review. 1998;5(2):173–196. 10.3758/BF03212943

[25] Wolfinger NH. Passing Moments: Some Social Dynamics of Pedestrian Interaction. J. of Contemporary Ethnography. 1995;24(3):323–340. 10.1177/089124195024003004

[26] Bascetta L, Ferretti G, Rocco P, Ardo H, Bruyninckx H, Demeester E, et al. Towards safe human-robot interaction in robotic cells: an approach based on visual tracking and intention estimation. In: Int. Conf. on Intelligent Robots and Systems; 2011. p. 2971–2978.

[27] Schulz D, Burgard W, Fox D, Cremers AB. Tracking multiple moving targets with a mobile robot using particle filters and statistical data association. In: Int. Conf. on Robotics and Automation. vol. 2; 2001. p. 1665–1670.

[28] Weinrich C, Volkhardt M, Einhorn E, Gross HM. Prediction of human collision avoidance behavior by lifelong learning for socially compliant robot navigation. In: Int. Conf. on Robotics and Automation (ICRA); 2013. p. 376–381.

[29] Kuderer M, Kretzschmar H, Sprunk C, Burgard W. Feature-Based Prediction of Trajectories for Socially Compliant Navigation. In: Robotics: Science and Systems; 2012.

[30] Arechavaleta G, Laumond J, Hicheur H, Berthoz A. The nonholonomic nature of human locomotion: a modeling study. In: Int. Conf. on Biomedical Robotics and Biomechatronics; 2006. p. 158–163.

[31] Puydupin-Jamin AS, Johnson M, Bretl T. A convex approach to inverse optimal control and its application to modeling human locomotion. In: Int. Conf. on Robotics and Automation; 2012. p. 531–536.

[32] Flash T, Hogan N. The coordination of arm movements: an experimentally confirmed mathematical model. The J. of Neuroscience. 1985;5(7):1688–1703. 4020415 10.1523/JNEUROSCI.05-07-01688.1985 PMC6565116

[33] Papadopoulos AV, Bascetta L, Ferretti G. A Comparative Evaluation of Human Motion Planning Policies. IFAC Proc. Volumes. 2014;47(3):12299–12304. 10.3182/20140824-6-ZA-1003.01898

[34] Ferrer G, Sanfeliu A. Comparative analysis of human motion trajectory prediction using minimum variance curvature. In: Int. Conf. on Human-Robot Interaction; 2011. p. 135–136.

[35] Hicheur H, Pham QC, Arechavaleta G, Laumond JP, Berthoz A. The formation of trajectories during goal-oriented locomotion in humans. I. A. stereotyped behaviour. European J. of Neuroscience. 2007;26(8):2376–2390. 10.1111/j.1460-9568.2007.05836.x 17953625

[36] Hoogendoorn S, Bovy P. Simulation of pedestrian flows by optimal control and differential games. Optimal Control Applications and Methods. 2003;24(3):153–172. 10.1002/oca.727

[37] Kruse T, Basili P, Glasauer S, Kirsch A. Legible Robot Navigation in the Proximity of Moving Humans. In: Int. Workshop on Advanced Robotics and its Social Impacts; 2012. p. 83–88.

[38] Kruse T, Kirsch A, Khambhaita H, Alami R. Evaluating Directional Cost Models in Navigation. In: Int. Conf. on Human-Robot Interaction; 2014. p. 350–357.

[39] Olivier AH, Marin A, Crétual A, Berthoz A, Pettré J. Collision avoidance between two walkers: Role-dependent strategies. Gait & Posture. 2013;38(4):751–756. 10.1016/j.gaitpost.2013.03.017 23665066

[40] Houska B, Ferreau HJ, Diehl M. ACADO toolkit—An open-source framework for automatic control and dynamic optimization. Optimal Control Applications and Methods. 2011;p. 298–312. 10.1002/oca.939

[41] Allgöwer G, Badgwell TA, Qin JS, Rawlings JB, Wright SJ. Nonlinear predictive control and moving horizon estimation—an introductory overview In: Advances in Control. Springer; 1999 p. 391–449.

[42] Fink PW, Foo PS, Warren WH. Obstacle avoidance during walking in real and virtual environments. Trans. on Applied Perception (TAP). 2007;4(1):2 10.1145/1227134.1227136

[43] Karamouzas I, Overmars M. A Velocity-Based Approach for Simulating Human Collision Avoidance In: Intelligent Virtual Agents. vol. 6356 of Lecture Notes in Computer Science. Springer; 2010 p. 180–186. 10.1007/978-3-642-15892-6_19

[44] Moussaïd M, Helbing D, Theraulaz G. How simple rules determine pedestrian behavior and crowd disasters. National Academy of Sciences. 2011;108(17):6884–6888. 10.1073/pnas.1016507108 PMC3084058 21502518

[45] Pham QC, Hicheur H. On the open-loop and feedback processes that underlie the formation of trajectories during visual and nonvisual locomotion in humans. J. of Neurophysiology. 2009;102(5):2800–2815. 10.1152/jn.00284.2009 19741106

[46] Wilkie RM, Wann JP, Allison RS. Active gaze, visual look-ahead, and locomotor control. J. of Experimental Psychology: Human Perception and Performance. 2008;34(5):1150 10.1037/0096-1523.34.5.1150 18823202

[47] Patla AE, Vickers JN. How far ahead do we look when required to step on specific locations in the travel path during locomotion? Experimental Brain Research. 2003;148(1):133–138. 10.1007/s00221-002-1246-y 12478404

[48] Hicheur H, Vieilledent S, Richardson M, Flash T, Berthoz A. Velocity and curvature in human locomotion along complex curved paths: a comparison with hand movements. Experimental Brain Research. 2005;162(2):145–154. 10.1007/s00221-004-2122-8 15586276

[49] Vieilledent S, Kerlirzin Y, Dalbera S, Berthoz A. Relationship between velocity and curvature of a human locomotor trajectory. Neuroscience Letters. 2001;305(1):65–69. 10.1016/S0304-3940(01)01798-0 11356309

[50] Zehr E, Duysens J. Regulation of arm and leg movement during human locomotion. The Neuroscientist. 2004;10(4):347–361. 10.1177/1073858404264680 15271262

[51] Buss M, Schmidt G. Control Problems in Multi-Modal Telepresence Systems In: Frank P, editor. Advances in Control. Springer London; 1999 p. 65–101.

[52] Olson PL, Sivak M. Perception-response time to unexpected roadway hazards. Human Factors: The J. of the Human Factors and Ergonomics Society. 1986;28(1):91–96. 3710489 10.1177/001872088602800110

[53] Stuart S, Alcock L, Galna B, Lord S, Rochester L. Re-test Reliability and Accuracy of the Dikablis Eye-Tracker when Sitting, Standing and Walking. In: World Congress of the Int. Society of Posture and Gait Research; 2014.

